# Supplementary material for: Conserved nucleocytoplasmic density homeostasis drives cellular organization across eukaryotes
Source: Nat Commun. 2025 Aug 15;16:7597. doi: 10.1038/s41467-025-62605-0 (PMC12356907; doi:10.1038/s41467-025-62605-0)
Supplement: Supplementary file 1 — Supplementary Information [file 41467_2025_62605_MOESM1_ESM.pdf]

Supplementary Information for

Conserved nucleocytoplasmic density homeostasis drives cellular organization across eukaryotes

Abin Biswas, Omar Muñoz, Kyoohyun Kim, Carsten Hoege, Benjamin M. Lorton, Rainer Nikolay, Matthew L. Kraushar, David Shechter, Jochen Guck, Vasily Zaburdaev, and Simone Reber

Corresponding authors: jochen.guck@mpl.mpg.de, vasily.zaburdaev@fau.de, reber@mpiib-berlin.mpg.de

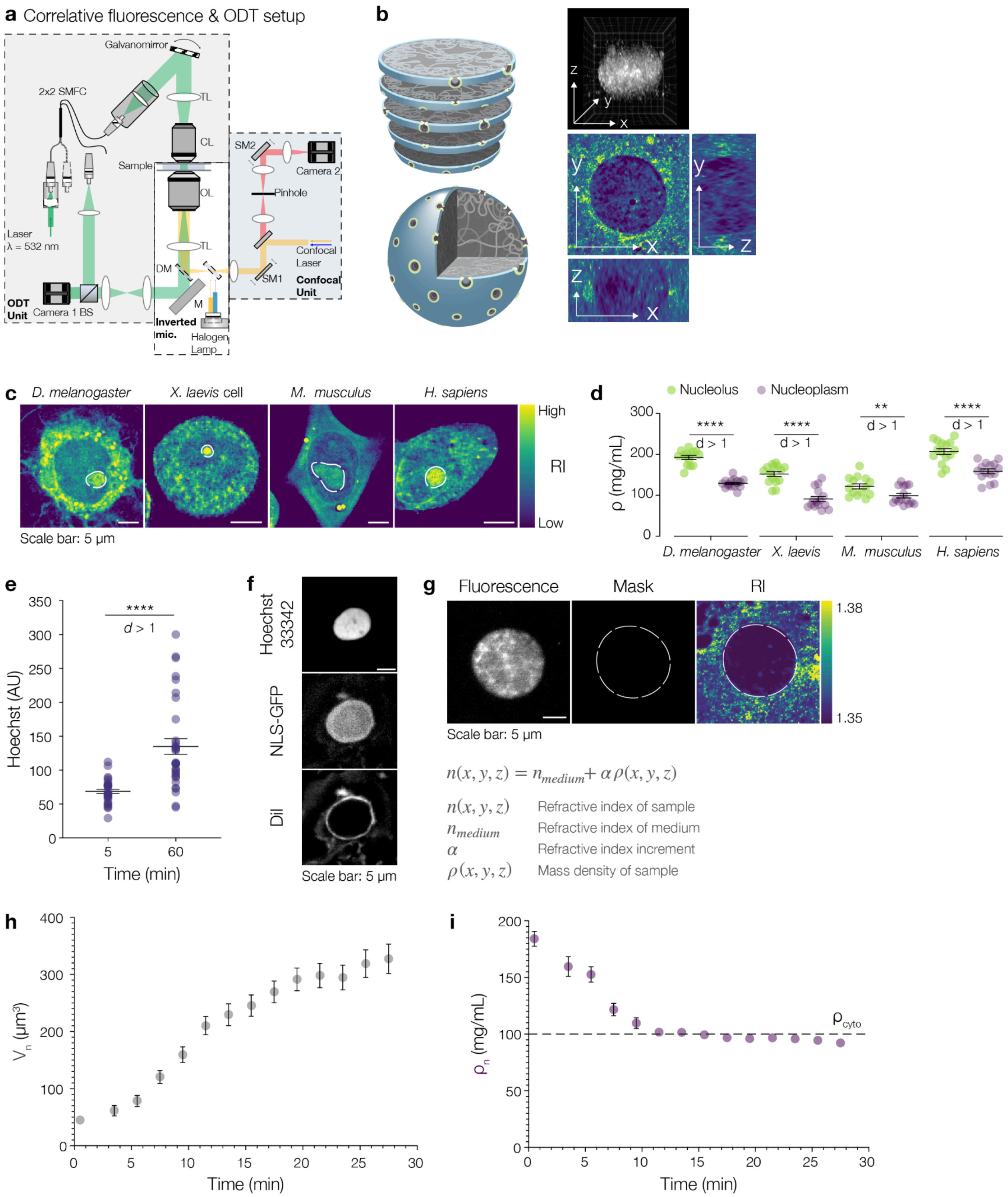

Supplementary Fig. 1. A combined optical diffraction tomography and confocal fluorescence microscopy setup provides volume and density information during nuclear assembly in *Xenopus* egg extracts

(**a**) Schematic of the optical setup used for experiments. SMFC, single mode fibre coupler; TL, tube lens; CL, condenser lens; OL, objective lens; M, mirror; DM, dichroic mirror; BS, beam splitter; SM1, scanning mirror 1; SM2, scanning mirror 2. See Methods for more details.

(**b**) Correlative confocal fluorescence and optical diffraction tomography (ODT) provides 3D volume and refractive index (RI) distributions.
(**c**) RI images of representative *D. melanogaster*, *X. laevis*, *M. musculus* and *H. sapiens* cells. The nucleolus has been marked with a white dashed line. The colored bar on the right shows the RI range. Scale bar: 5 μm.
(**d**) Quantification of nucleolar (green) and nucleoplasmic (purple) density in each cell type. Each symbol represents the average value from the segmented nucleolus or nucleoplasm.
(**e**) Nuclei assembled in *Xenopus* extracts are replication competent as indicated by the quantification of total Hoechst-33342 intensity at 5 minutes and 60 minutes.
(**f**) Nuclei assembled in *Xenopus* egg extract are import competent. Representative fluorescence image where DNA was stained with Hoechst-33342, NLS-GFP was used to show active import and membranes were stained with DiI. All scale bars: 5 μm.
(**g**) Representative fluorescence image of a *Xenopus* nucleus (stained with Hoechst-33342) at 60 min and corresponding mask obtained by segmentation. Masks were obtained from the fluorescence images and transferred to the ODT images to quantify the mass density of regions of interest. Color bar on the right shows the RI range in the ODT image. Equation used to obtain the mass density (ρ) of the sample from the 3D RI using the refractive index increment α. See Methods for more details.
(**h**) Quantification of nuclear volume (V_n) at 2 min time intervals as nuclei assemble. Symbols show mean values.
(**i**) Quantification of nuclear mass density (ρ_n) at 2 min time intervals as nuclei assemble. Black dashed line shows the mass density of the surrounding cytoplasm (ρ_{cyto}). For (**h**) and (**i**), circles represent the mean. Black lines and bars in all graphs represent the mean ± SEM. Source data are provided as a Source Data file. Sample size and replicate information available in the Statistics and Reproducibility section.

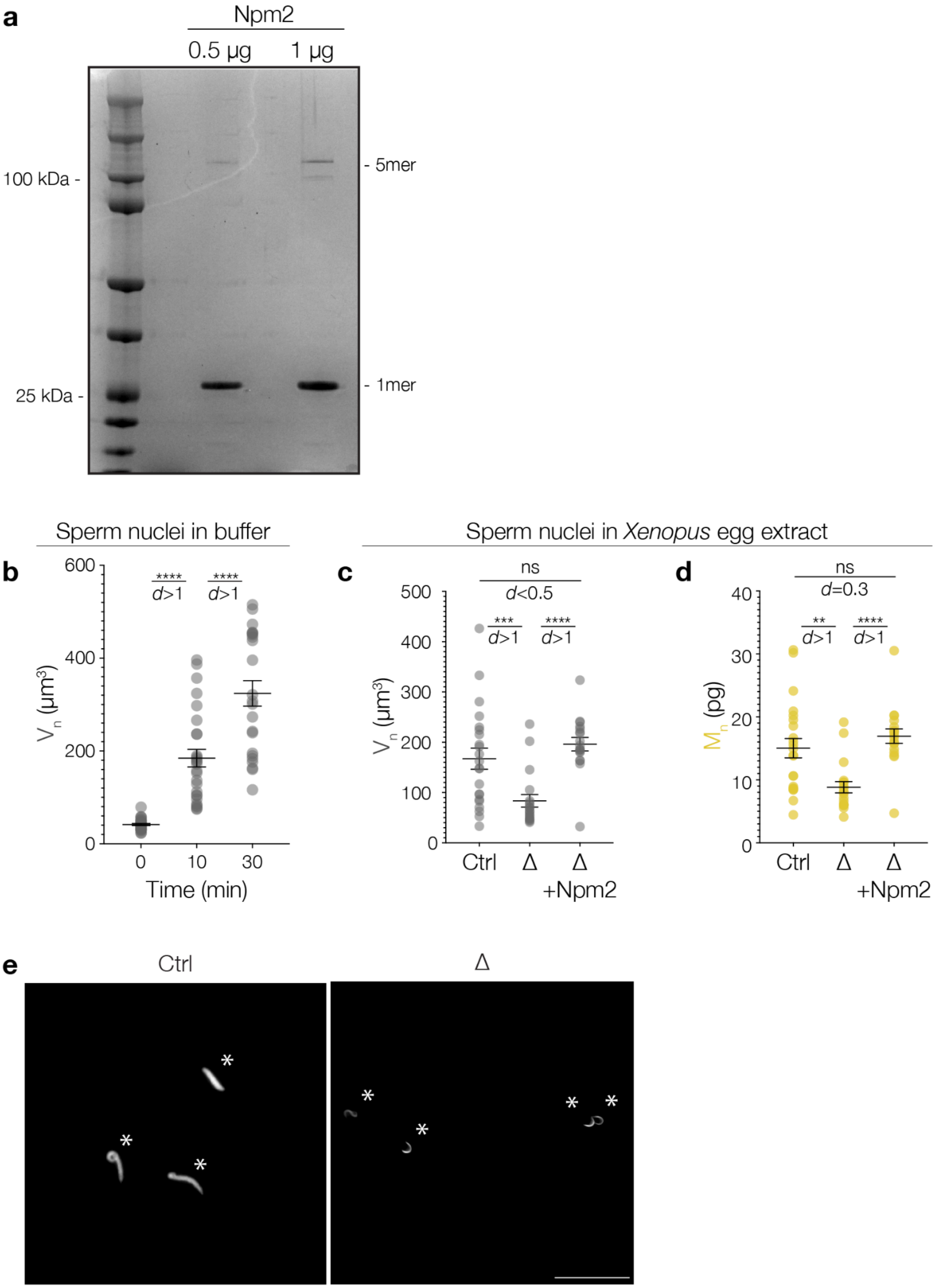

Scale bar: 50 µm

Supplementary Fig. 2. Nucleoplasmin is both necessary and sufficient to reduce nuclear density to that of the surrounding solvent.
(**a**) Coomassie stain of an SDS gel showing purified Nucleoplasmin (Npm2) protein. Two distinct bands can be seen for the pentameric and monomeric form of the protein. 0.5 µg and 1 µg of protein were loaded. Left most lane shows the molecular weight marker.
(**b**) Chromatin volume (V_n, grey) at 0, 10, and 30 minutes after the addition of Npm2. Each symbol represents the value from one decondensing sperm nucleus.
(**c**) Chromatin volume in control-depleted, ΔNpm2 and Δ+Npm2 extracts. After 10 minutes, sperm nuclei in ΔNpm2 extracts have a lower volume than decondensing sperm nuclei in control-depleted and Δ+Npm2 extracts. There is no significant difference between the volume of sperm nuclei in control-depleted and Δ+Npm2 extracts.
(**d**) Quantification of dry mass (M_n, yellow) in control-depleted, ΔNpm2 and Δ+Npm2 extracts. After 10 minutes, sperm nuclei in ΔNpm2 extracts have a lower dry mass than decondensing sperm nuclei in control-depleted and Δ+Npm2 extracts. There is no significant difference between the dry mass of sperm nuclei in control-depleted and Δ+Npm2 extracts.
(**e**) Low magnification fluorescence images showing sperm nuclei (asterisks) stained with Hoechst-33342 in control-depleted and ΔNpm2 extracts after 10 minutes of incubation. Scale bar: 50 µm.
Black lines and bars in all graphs represent the mean ± SEM. Source data are provided as a Source Data file. Sample size and replicate information available in the Statistics and Reproducibility section.

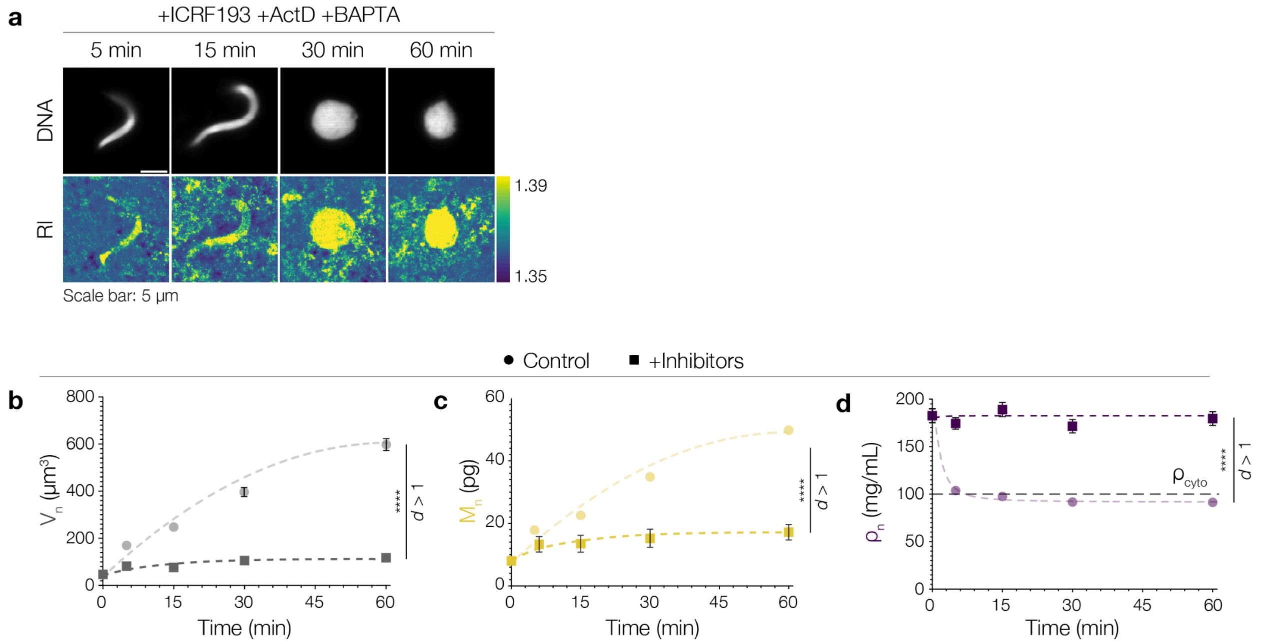

Supplementary Fig. 3. Inhibition of chromatin decondensation and nuclear import leaves nuclei with a density comparable to that of fully condensed sperm chromatin.

(**a**) Nuclei were assembled in *Xenopus* egg extracts in the presence of ICRF193 (a topoisomerase inhibitor), Actinomycin-D (ActD, a DNA intercalator) and BAPTA (a calcium chelator). This inhibitor cocktail inhibits chromatin decondensation and allows for poreless nuclear envelope closure*. Top panel shows representative fluorescence images of Hoechst-33342 stained nuclei (DNA) from different time points. Bottom panel shows the corresponding RI image. While assembling nuclei round up, they have a RI value comparable to that of fully condensed sperm chromatin.

(**b**) Volume (V_n, grey) of poreless nuclei. Symbols represent the mean values. In **b-d**, circles and lighter colors show the values for the control nuclei while squares and darker colors show the values for perturbed nuclei. At 60 minutes there is a significant difference between the volumes of nuclei. In **b-d**, dashed lines are used to guide readers along the trend followed by the experimental data.

(**c**) Dry mass (M_n, yellow) of poreless nuclei. For poreless nuclei, M_n does not change during nuclear assembly.

(**d**) Density of poreless nuclei (ρ_n, purple). For poreless nuclei, ρ_n does not change during nuclear assembly. Black dashed line: ρ of the cytoplasm.

Black lines and bars in all graphs represent the mean ± SEM. Source data are provided as a Source Data file. Sample size and replicate information available in the Statistics and Reproducibility section.

* Macaulay, C., & Forbes, D. J. (1996). Assembly of the nuclear pore: biochemically distinct steps revealed with NEM, GTP gamma S, and BAPTA. *The Journal of cell biology*, *132*(1), 5-20.

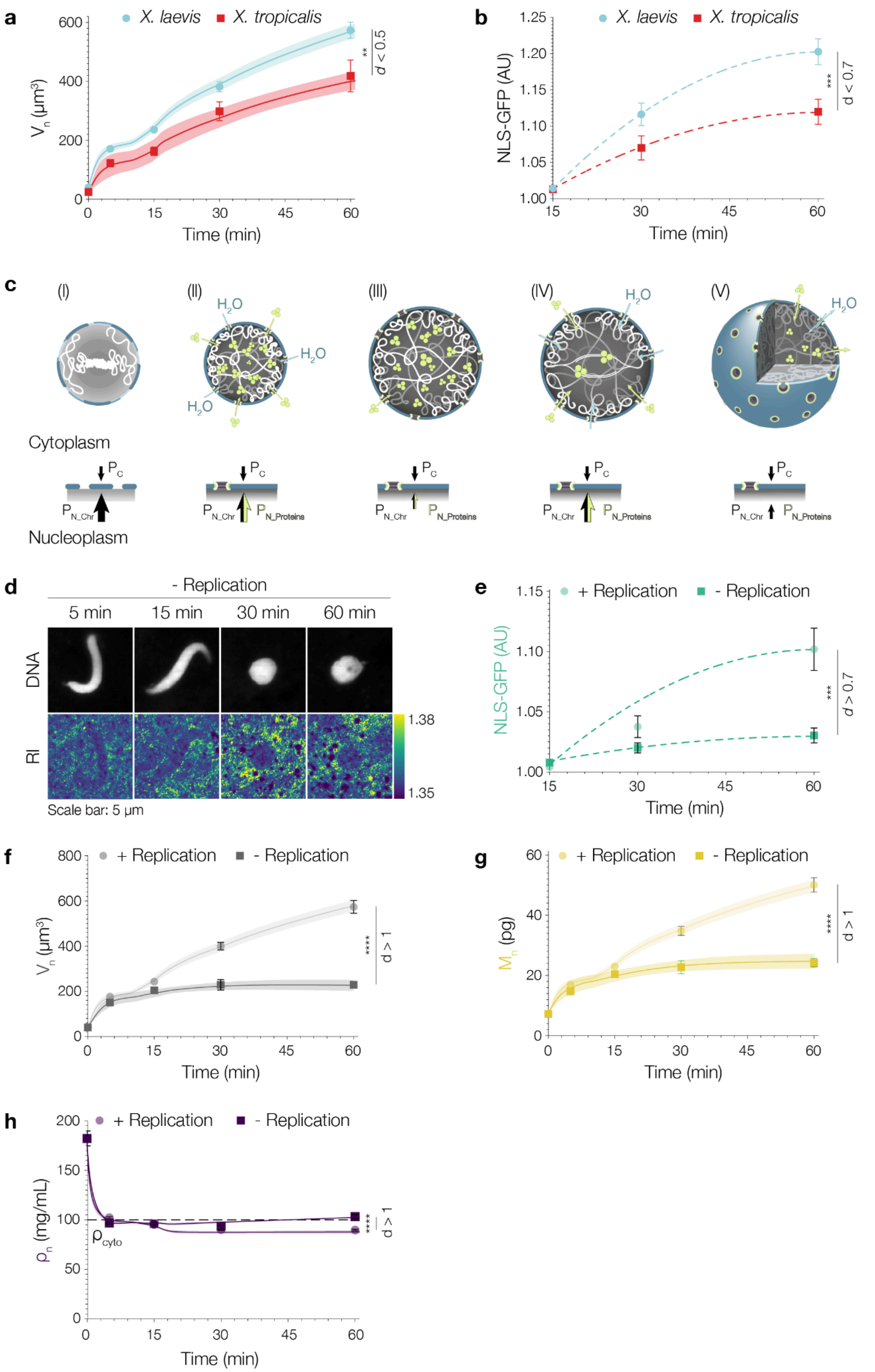
a
X. laevis
X. tropicalis
V_n (µm³)
Time (min)
**
d < 0.5
b
NLS-GFP (AU)
Time (min)
***
d < 0.7
c
(I)
(II)
(III)
(IV)
(V)
H2O
Cytoplasm
Nucleoplasm
P_C
P_N_Chr
P_N_Proteins
d
- Replication
5 min
15 min
30 min
60 min
DNA
RI
1.38
1.35
Scale bar: 5 µm
e
+ Replication
- Replication
NLS-GFP (AU)
Time (min)
***
d > 0.7
f
+ Replication
- Replication
V_n (µm³)
Time (min)
****
d > 1
g
+ Replication
- Replication
M_n (pg)
Time (min)
****
d > 1
h
+ Replication
- Replication
ρ_n (mg/mL)
ρ_cyto
Time (min)
****
d > 1

Supplementary Fig. 4. **Osmotically active solute macromolecules determine nuclear density and volume.**

(**a**) Volume (V_n) at different time points for nuclei assembled using *X. laevis* sperm (blue circles) and *X. tropicalis* sperm (red squares) in *X. laevis* extract. *X. tropicalis* nuclei are approx. 30% smaller than *X. laevis* nuclei. Symbols represent the mean values. In panels **a** and **f-h**, bold lines and shaded areas represent the mean ± SEM from the theoretical simulations (Supplementary Note - Theory).

(**b**) Quantification of NLS-GFP intensity at different time points of nuclear assembly. *X. laevis* nuclei are more import efficient in comparison to *X. tropicalis* nuclei. Dashed lines in **b & e** are used to guide readers along the trend followed by the experimental data.

(**c**) Pressure balance model. (I) Chromatin unfolds towards its thermodynamically preferred volume in the cytoplasm. (II) At the same time, nuclear envelope assembly starts and confines the chromatin to a well-defined nuclear compartment, which creates an outward pressure. Then, nucleocytoplasmic transport, with rates dependent on chromatin content, establishes the nuclear identity with a net accumulation of nuclear proteins. As a consequence, the nuclear dry mass increases. The imported nuclear protein complexes create an outward colloid osmotic pressure, which — assisted by chromatin pressure — drives water influx into the nucleus and thus nuclear growth (III). (IV) Replicating chromatin contributes to the pressure balance both directly as a confined polymer-like chain and indirectly as a regulator of nuclear import, and thus drives nuclear growth further (V).

(**d**) Nuclear assembly in the presence of the replication inhibitor Aphidicolin. Top row shows fluorescence images of DNA stained with Hoechst-33342 and bottom row shows the RI distribution. Color bar on the right shows the RI range. Scale bar: 5 µm.

(**e**) Replication competent nuclei (+ Replication, circles) are more import efficient in comparison to perturbed nuclei (– Replication, squares). Quantification of NLS-GFP intensity at different time points of nuclear assembly. Dashed lines are used to guide readers along the trend followed by the experimental data. In **e-h**, circles and lighter colors show the values for + Replication nuclei while squares and darker colors show the values for - Replication nuclei.

(**f**) Blocking replication reduces nuclear volume (V_n). Quantification of V_n at different time points after the start of nuclear assembly.

(**g**) Blocking replication reduces nuclear dry mass (M_n). Quantification of M_n at different time points of nuclear assembly.

(**h**) Blocking replication inhibits decrease in nuclear density (ρ_n). Quantification of ρ_n at different time points after the start of nuclear assembly. Cytoplasmic density indicated by black line (ρ_{cyto}).

Black lines and bars in all graphs represent the mean ± SEM. Source data are provided as a Source Data file. Sample size and replicate information available in the Statistics and Reproducibility section.

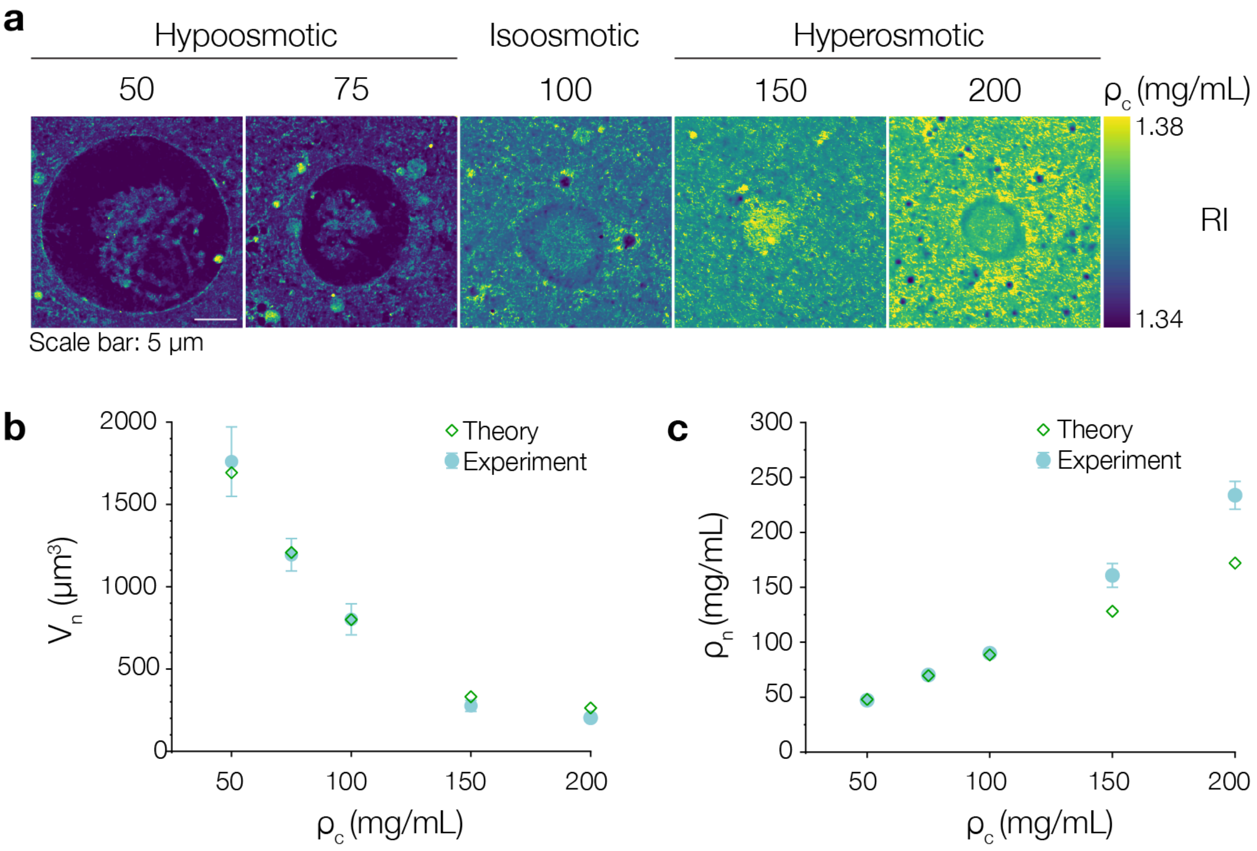

Supplementary Fig. 5. Nuclear volume changes in response to total protein density.
(**a**) Nuclei alter their volume in response to changes in total protein density. Representative RI images of nuclei assembled in *Xenopus* egg extract with reducing protein density by dilution with CSF-XB buffer (Hypoosmotic) or increasing protein density by adding BSA (Hyperosmotic). Undiluted extract has a protein concentration of 100 ± 2 mg/mL. Scale bar: 5 μm. Bar shows RI distribution range.
(**b**) Quantification of nuclear volumes (V_n) in response to changing protein density. Experimental data shown in blue circles (mean ± SEM) and theoretical predictions as green diamonds.
(**c**) Quantification of nuclear density (ρ_n) in response to changing protein density. Experimental data shown in blue circles (mean ± SEM) and theoretical predictions as green diamonds.
Source data are provided as a Source Data file. Sample size and replicate information available in the Statistics and Reproducibility section.

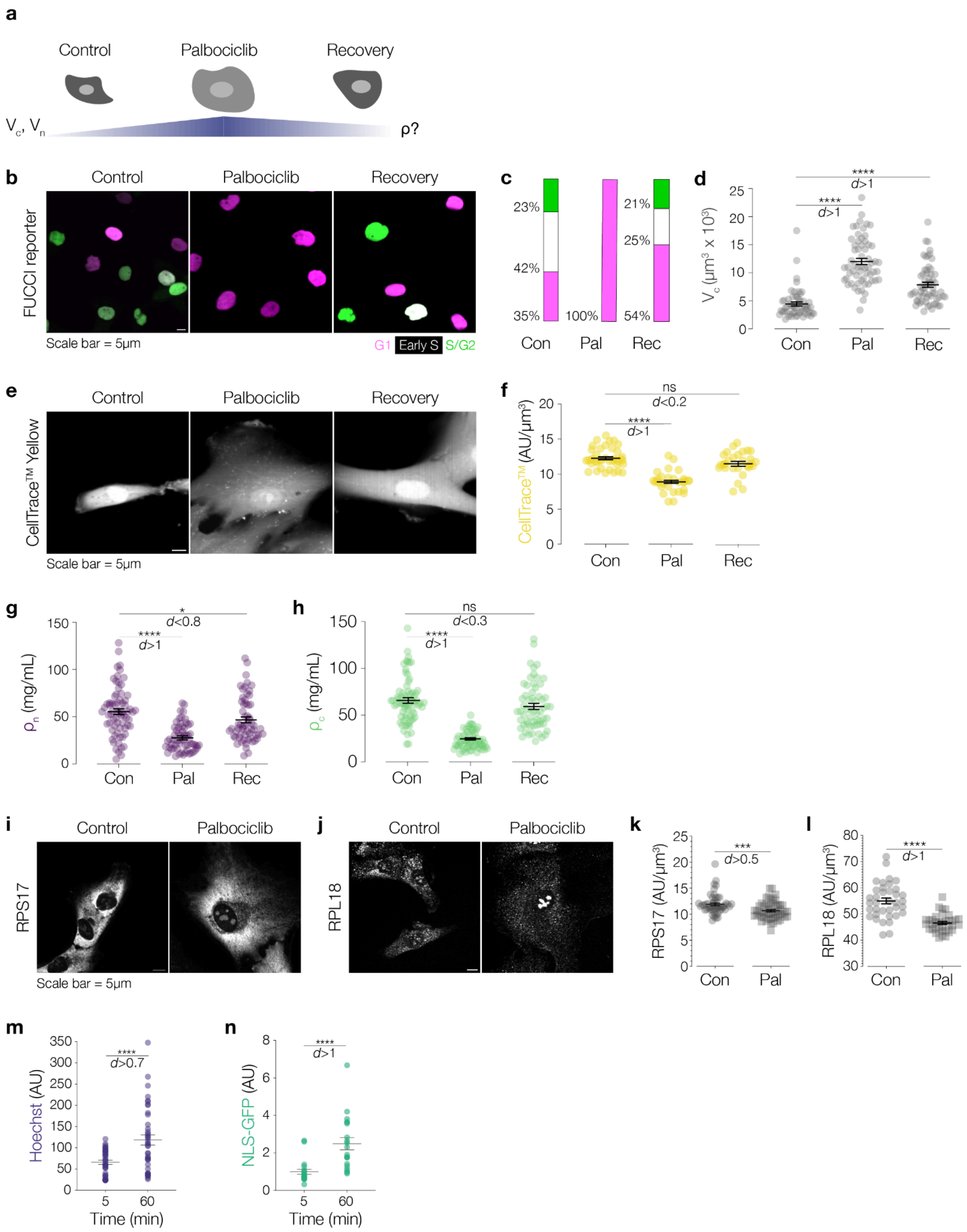

Supplementary Fig. 6. Reversal of the NC density ratio during senescence and *in vitro* is linked to heavy osmotically inactive cytoplasmic components.

(**a**) Schematic representing the senescence induction and recovery experiments.

(**b**) Upon Palbociclob treatment, cells arrest in G1. Representative images of hTERT RPE1-FUCCI cells in different stages of the cell cycle. Pink nuclei indicate cells in the G1 phase, white nuclei indicate cells in early S phase and green nuclei indicate cells in the S/G2 phase of the cell cycle. All scale bars: 5 µm.
(**c**) Quantification of cell cycle phase in different conditions.
(**d**) Cell volume increases upon senescence induction. Quantification of cell volume (V_n, grey) in the different conditions. Of note, recovering cells remain large in comparison to control cells.
(**e**) Representative images of live cells stained with CellTrace™ Yellow total protein stain.
(**f**) Quantification of protein concentration using CellTrace™ Yellow (total fluorescence intensity per cell/ cell volume) in the different conditions. Protein concentration reduces during senescence (Pal) but recovers (Rec) upon inhibitor removal.
(**g**) Nuclear density as measured by ODT reduces upon senescence induction. Quantification of nuclear density (ρ_n) upon senescence induction and recovery.
(**h**) Cytoplasmic density as measured by ODT reduces upon senescence induction. Quantification of nuclear density (ρ_{cyto}) upon senescence induction and recovery.
(**i-j**) Representative immunofluorescence images showing the distribution of pre-ribosomal subunits (**i**) RPS17 and (**j**) RPL18 in control and senescent cells (Palbociclib).
(**k-l**) Quantification of cellular (**k**) RPS17 and (**l**) RPL18 concentrations during senescence. Both concentrations reduce upon Palbociclib treatment.
(**m**) Nuclei assembled in high-speed (HS) *Xenopus* extract are replication competent. Quantification of total Hoechst-33342 intensity at 5 min and 60 minutes after starting the assembly reaction.
(**n**) Nuclei assembled in HS *Xenopus* extract are import competent. Quantification of total GFP intensity 5 min and 60 minutes after starting the assembly reaction.
Black bars and lines indicate mean ± SEM. Black dashed line. Source data are provided as a Source Data file. Sample size and replicate information available in the Statistics and Reproducibility section.

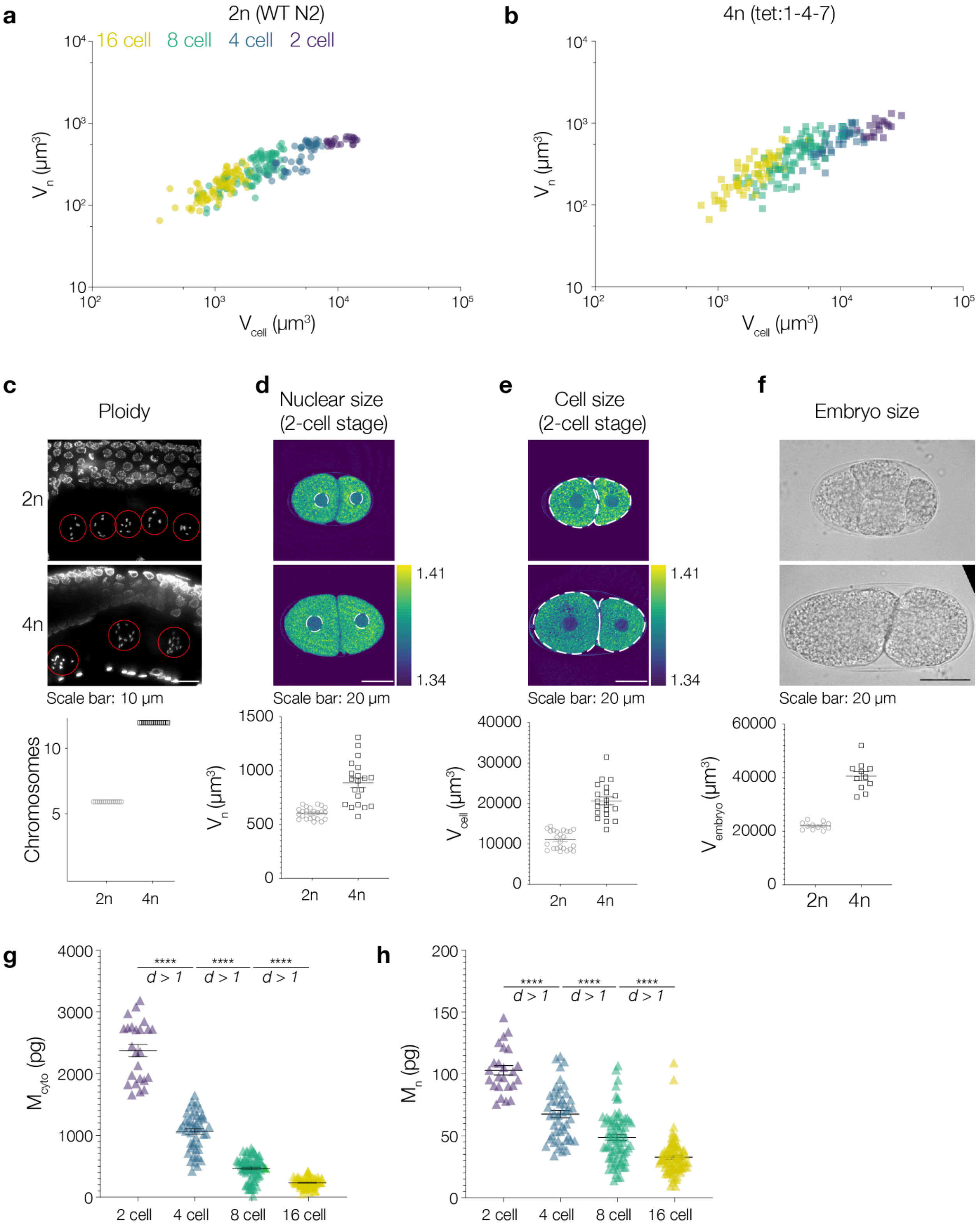

Supplementary Fig. 7. Hierarchical size scaling in *C. elegans*.
(**a**) Double logarithmic plot of nuclear volume versus cell volume in wildtype diploid and (**b**) tetraploid *C. elegans* embryos. Purple, blue, green, and yellow circles represent values from 2, 4, 8 and 16 cell stage cells, respectively.

(**c**) Hoechst-33342 stained nuclei (red circles) from wildtype (top) and tetraploid (bottom) gonads. Scale bar: 10 µm. Quantification of chromosome number in 1-4-7 worms confirmed tetraploidy for all.
(**d**) Nuclei in tetraploid embryos have a larger volume than nuclei in wildtype embryos. Representative ODT image from a 2-stage wildtype and tetraploid embryo. White dashed lines show nuclear boundaries. Color bar on the right shows the refractive index range. Scale bar: 20 µm.
(**e**) Cells in tetraploid embryos have a larger volume than cells in wildtype embryos. Representative ODT image from a 2-cell wildtype and tetraploid embryo. White dashed lines show cell boundaries. Color bar on the right shows the refractive index range.
(**f**) Tetraploid embryos are larger than wildtype embryos. Brightfield images of a 4-cell stage wildtype embryo and a 2-cell stage tetraploid embryo.
(**g**) Cytoplasmic and (**h**) nuclear dry mass reduces during development in wildtype embryos.
Black lines and bars in all graphs represent the mean ± SEM. Source data are provided as a Source Data file. Sample size and replicate information available in the Statistics and Reproducibility section.

Statistics and Reproducibility

In the following section, details about the number of samples, independent experiments, statistical tests and significance values are provided. No statistical test was used to predetermine sample size. No data were excluded from the analyses. All experiments were replicated independently, the number of replicates has been indicated below. Cells and nuclei were imaged randomly. No blinding was carried out as all measurements were quantitative and were not biased by subjective perception.

Supplementary Figure 1

(**d**) n = 15 cells (75 ROIs per species, 5 ROIs per cell). Mann Whitney test was used to test for statistical significance. p value *Drosophila melanogaster* nucleus & nucleolus < 0.0001, p value *Xenopus laevis* nucleus & nucleolus < 0.0001, p value *Mus musculus* nucleus & nucleolus = 0.0086 and p value *Homo sapiens* nucleus & nucleolus < 0.0001. In addition to the significance, we also indicate the effect size by calculating Cohen's *d*. An effect size between 0.20 – 0.50 was considered small, while effect sizes between 0.51 – 0.80 were considered medium and d > 0.81 was considered large.

(**e**) n = 31 nuclei at 5 min and n = 33 nuclei at 60 min from 3 independent experiments. Mann Whitney test was used to test for statistical significance. p value 5 min & 60 min < 0.0001.

(**h & i**) n = 17 nuclei for 0 min, n = 22 nuclei for 3 min, n = 38 nuclei for 5-11 min and 17-21 min, n = 37 nuclei for 13-15 min and 23 min, and n = 31 nuclei for 25 and 27 min.

Supplementary Figure 2

(**b**) n = 30 nuclei at 0 min, n = 27 nuclei at 10 min and n = 23 nuclei at 30 min from 3 independent experiments. Mann Whitney test was used to test for statistical significance. p value 0 & 10min < 0.0001 , p value 10 & 30 min < 0.0001. In addition to the significance, we also indicate the effect size by calculating Cohen's *d*. An effect size between 0.20 – 0.50 was considered small, while effect sizes between 0.51 – 0.80 were considered medium and d > 0.81 was considered large.

(**c**) n = 22 nuclei for Ctrl, n = 19 nuclei for Δ and n = 18 nuclei for Δ+Npm2 from 3 independent experiments. Mann Whitney test was used to test for statistical significance. p value Ctrl & Δ = 0.0010, p value Δ & Δ+Npm2 < 0.0001 and Ctrl & Δ+Npm2 = 0.1472.

(**d**) n = 22 nuclei for Ctrl, n = 19 nuclei for Δ and n = 18 nuclei for Δ+Npm2 from 3 independent experiments. Mann Whitney test was used to test for statistical significance. p value Ctrl & Δ = 0.0012, p value Δ & Δ+Npm2 < 0.0001 and Ctrl & Δ+Npm2 = 0.2188.

Supplementary Figure 3

(**b, c & d**) n = 20 nuclei from 2 independent experiments. Mann Whitney test was used to test for statistical significance. p value Control & +Inhibitors < 0.0001. In addition to the significance, we also indicate the effect size by calculating Cohen's *d*. An effect size

between 0.20 – 0.50 was considered small, while effect sizes between 0.51 – 0.80 were considered medium and d > 0.81 was considered large.

Supplementary Figure 4

(**a**) n = 15 nuclei for 0 min, n = 25 nuclei for 5 min, n = 29 nuclei for 15 min, n = 29 nuclei for 30 min and n = 30 nuclei for 60 min from 3 independent experiments. Mann Whitney test was used to test for statistical significance. p value Laevis & Trops = 0.0057. In addition to the significance, we also indicate the effect size by calculating Cohen's *d*. An effect size between 0.20 – 0.50 was considered small, while effect sizes between 0.51 – 0.80 were considered medium and d > 0.81 was considered large.

(**b**) For *X. laevis*, n = 32 nuclei for 15 min, n = 34 nuclei for 30 min and n = 35 nuclei for 60 min from 3 independent experiments. For *X. tropicalis*, n = 28 nuclei for 15 min, n = 28 nuclei for 30 min and n = 29 nuclei for 60 min from 3 independent experiments. Mann Whitney test was used to test for statistical significance. p value *X. laevis* & *X. tropicalis* = 0.0005.

(**e**) n = 30 nuclei for both conditions from 3 independent experiments. Mann Whitney test was used to test for statistical significance. p value + Replication & - Replication = 0.0008.

(**f, g & h**) n = 29 nuclei for 5 min, n = 29 nuclei for 15 min, n = 26 nuclei for 30 min and n = 31 nuclei for 60 min from 3 independent experiments. Mann Whitney test was used to test for statistical significance. p value + Replication & - Replication < 0.0001.

Supplementary Figure 5

(**b & c**) n = 30 nuclei for 50 mg/mL, n = 20 nuclei for 75 mg/mL, n = 45 nuclei for 100 mg/mL, n = 10 nuclei for 150 mg/mL and n = 15 nuclei for 200 mg/mL from 3 independent experiments.

Supplementary Figure 6

(**c**) n = 52 cells for Con, n = 51 cells for Pal and n = 59 cells for Rec from 3 independent experiments.

(**d**) n = 60 cells from 3 independent experiments. Mann Whitney test was used to test for statistical significance. p value Con & Pal < 0.0001 and p value Con & Rec < 0.0001. In addition to the significance, we also indicate the effect size by calculating Cohen's *d*. An effect size between 0.20 – 0.50 was considered small, while effect sizes between 0.51 – 0.80 were considered medium and d > 0.81 was considered large.

(**f**) n = 30 cells from 3 independent experiments. Mann Whitney test was used to test for statistical significance. p value Con & Pal < 0.0001 and p value Con & Rec = 0.4761.

(**g**) n = 60 cells from 3 independent experiments. Mann Whitney test was used to test for statistical significance. p value Con & Pal < 0.0001 and p value Con & Rec = 0.0142.

(**h**) n = 60 cells from 3 independent experiments. Mann Whitney test was used to test for statistical significance. p value Con & Pal < 0.0001 and p value Con & Rec = 0.0590.

(**k**) n = 53 cells for Con and n = 54 cellsfor Pal from 3 independent experiments. Mann Whitney test was used to test for statistical significance. p value Con & Pal = 0.0004.

(**l**) n = 37 cells for Con and n = 36 cells for Pal from 3 independent experiments. Mann Whitney test was used to test for statistical significance. p value Con & Pal < 0.0001.
(**m**) n = 37 nuclei for 5 min and n = 40 nuclei for 60 min. Mann Whitney test was used to test for statistical significance. p value 5 min & 60 min < 0.0001.
(**n**) n = 20 nuclei from 2 independent experiments. Mann Whitney test was used to test for statistical significance. p value 5 min & 60 min < 0.0001.

Supplementary Figure 7
(**a**) n = 24 nuclei and cells for 2 cell stage, n = 48 nuclei and cells for 4 cell stage, n = 79 nuclei and cells for 8 cell stage and n = 85 nuclei and cells for 16 cell stage for wildtype (2n) embryos.
(**b**) n = 20 nuclei and cells for 2 cell stage, n = 44 nuclei and cells for 4 cell stage, n = 80 nuclei and cells for 8 cell stage and n = 58 nuclei and cells for 16 cell stage or tet: 1-4-7 (4n) embryos.
(**c**) n = 15 embryos from 3 independent experiments.
(**d & e**) n = 24 nuclei/cells for 2n and n = 20 nuclei/cells for 4n embryos from 3 independent experiments.
(**f**) n = 12 embryos for 2n and n =10 embryos for 4n from 3 independent experiments.
(**g**) n = 24 cells for 2 cell stage, n = 48 cells for 4 cell stage, n = 79 cells for 8 cell stage and n = 85 cells for 16 cell stage for wildtype (2n) embryos. Mann Whitney test was used to test for statistical significance. p value 2 cell & 4 cell < 0.0001, p value 4 cell & 8 cell < 0.0001, p value 8 cell & 16 cell < 0.0001. In addition to the significance, we also indicate the effect size by calculating Cohen's *d*. An effect size between 0.20 – 0.50 was considered small, while effect sizes between 0.51 – 0.80 were considered medium and d > 0.81 was considered large.
(**h**) n = 24 nuclei for 2 cell stage, n = 48 nuclei for 4 cell stage, n = 79 nuclei for 8 cell stage and n = 85 nuclei for 16 cell stage for wildtype (2n) embryos. Mann Whitney test was used to test for statistical significance. p value 2 cell & 4 cell < 0.0001, p value 4 cell & 8 cell < 0.0001, p value 8 cell & 16 cell < 0.0001.

Supplementary Table 1. DNA content, nuclear mass and volume in different species.

Species	DNA (haploid)	Chromosome number (haploid)	Ploidy	DNA mass (pg)	Nuclear volume (μm^3)	Nuclear density ($pg/\mu m^3$)	Nuclear mass (pg)	DNA mass / nuclear mass (%)
S. cerevisiae	12 Mb[a]	16[a]	2N	0.012	3.3[o]	0.31*	0.90	2.66
S. pombe	14 Mb[b]	3[j]	N	0.014	7.5*	0.18*	1.37	1.02
C. reinhardtii	110 Mb[c]	17[k]	N	0.11	34*	0.22*	7.55	1.46
C. elegans	100 Mb[d]	6[l]	2N	0.1	605* (2 cell)	0.17*	102.25	0.20
D. melanogaster	180 Mb[e]	4[m]	2N	0.18	292*	0.11*	33.29	1.08
X. laevis	3.1 Gb[f]	18[f]	2N**	3.1	573*	0.09*	49.85	12.43
X. tropicalis	1.7 Gb[f]	10[f]	2N	1.7	418*	0.09*	36.39	9.34
D. rerio	1.4 Gb[g]	25[n]	2N	1.4	840*	0.20*	165.48	1.70
M. musculus	2.5 Gb[h]	20[h]	2N	2.5	630*	0.11*	68.67	7.28
H. sapiens	3.4 Gb[i]	23[i]	2N	3.4	690*	0.16*	107.64	6.32

* values obtained from this study.
** Allotetraploid

a. Goffeau, A. et al. Life with 6000 genes. Science 274, 546, 563–7 (1996).
b. GenBank Sample ID: SAMEA3138176
c. Ensembl taxonomy id: 3055
d. Hodgkin, J., Plasterk, R. H. & Waterston, R. H. The nematode Caenorhabditis elegans and its genome. Science 270, 410–414 (1995).
e. Adams, M. D. et al. The genome sequence of Drosophila melanogaster. Science 287, 2185–2195 (2000).
f. Xenbase
g. BIONUMBERS ID: 111374
h. GenBank ID: GCF_000001635.27
i. GenBank ID: GCF_000001405.40
j. Wood, V. et al. The genome sequence of Schizosaccharomyces pombe. Nature 415, 871–880 (2002).
k. UniProt UP000006906
l. NCBI genome id: 41
m. NCBI genome id: 47
n. GenBank Sample ID: GCF_000002035.6
o. BIONUMBERS id:101396

Supplementary Notes

1. Theoretical Model of Nuclear Growth

Here, we present a model for nuclear growth starting from sperm chromatin in *X. laevis* egg extract until the end of one cycle of DNA replication, with the goal of correctly predicting nuclear mass density ρ_n. It uses realistic biophysical parameters and is able to reproduce the time evolution of the nuclear volume V_n and dry mass M_n in normal conditions and various perturbation experiments. Supplementary Notes Fig. 1 provides a graphical summary of our experimental observations made in Figs. 1 d-g and 2a of the main text: nuclear assembly and growth can be divided into two phases, which are formally separated by the closure of the nuclear envelope. Phase 1 represents the rapid decondensation of sperm chromatin by nucleoplasmin, while phase 2 shows more gradual dynamics where nucleocytoplasmic transport of proteins and DNA replication, via osmotic and chromatin pressures respectively, drive nuclear growth.

To predict nuclear mass density, the model focuses on two aspects: (i) determining nuclear volume V_n based on the balance of involved pressures for the experimentally determined protein content in the nucleoplasm and cytoplasm at fixed time points, and ii) describing the dynamic nuclear dry mass M_n as a result of nuclear transport, thus recapitulating the complete time-dependent nuclear growth.

We first detail the concept of pressure balance that is naturally applicable in phase 2 of nuclear growth, where the nuclear compartment is well defined. We discuss the idea of protein colloid osmotic pressure and chromatin pressure, as well as the role of protein complexes and protein identities. By using the pressure balance concept, we can recapitulate the experimental observations on nuclear volume and mass (section 1.1). We then extend this concept to phase 1 of nuclear assembly which helps us to define the effect of the excluded volume of chromatin (section 1.1.5). In phase 1 and 2, chromatin experiences changes first due to decondensation and then due to replication, respectively, which we describe as a function of time (section 1.1.6). We next explain how the nuclear protein content evolves over time due to active nuclear import and quantify it by means of

the kinetic model (section 1.2). By combining the concept of pressure balance with nuclear import and taking time-dependent parameters (chromatin decondensation and content) into account, we provide a complete dynamical model of nuclear growth linking both phases (section 1.2.2). In the concluding sections 1.3, 1.4, and 1.5 we explain the choice of model parameters, demonstrate the validity of the model for perturbation experiments and provide technical details of model implementation, respectively.

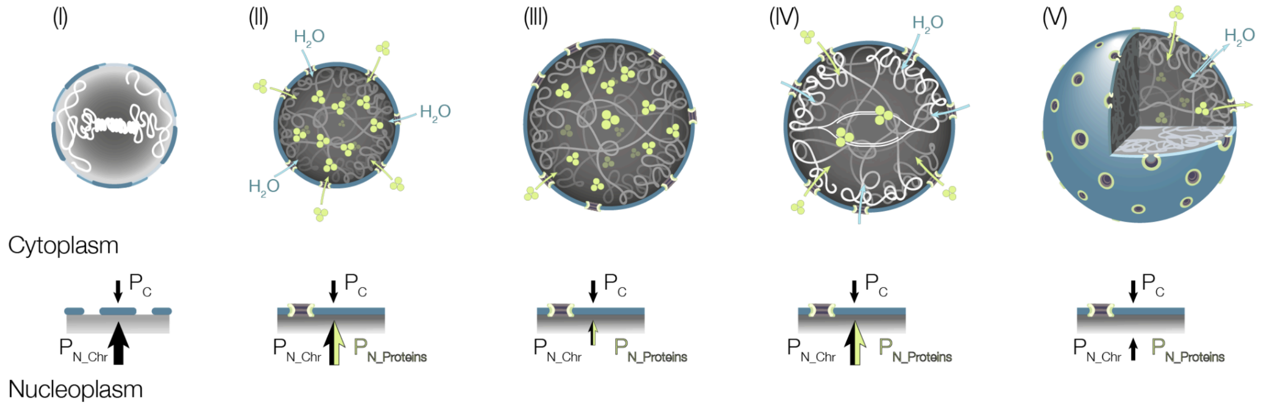

Supplementary Notes Fig. 1 | Summary of essential processes happening during nuclear growth. Decondensation of sperm chromatin driven by nucleoplasmin leads to its unfolding while the nuclear envelope forms [(I), $t = 0 - 5$ min]. The fully functional nuclear envelope marks the beginning of phase 2 [(II), $t = 10 - 15$ min]. Specific proteins are imported and the volume of the nucleus grows (III). DNA replication starts at around $t = 20 - 30$ min (IV) and is finished by $t = 60$ min (V).

Before going into the details of our specific model, we would like to clarify how it is an extension of the Pump-Leak model, which has previously been used by various authors to describe systems such as eukaryotic cells [1–3]. The Pump-Leak model is a coarse-grained model, which in its simplest form, considers the mechanical and osmotic balances of a compartment with the external medium. Imbalances lead to water transport into or outside of the compartment and results in changes to the compartment volume.

In order to predict the dry mass density of the compartment, we couple the Pump-Leak model to a model that determines the compartment's dry mass. For our experimental system, the compartment is the nucleus and the external medium is the cytoplasmic extract. The increase in nuclear dry mass is caused by active import of

proteins from the extract into the nucleus. The timescale for this process is in the order of several minutes (see e.g Fig. 1d-g) and is much slower than the timescale at which water equilibrates across the nuclear membrane, which is in the order of a few seconds [3]. Therefore, we can assume that water equilibration happens instantaneously [3–5], which leads to the pressure balance across the nuclear envelope (see [3] for a more detailed derivation).

Taken together, our model can be described as a quasi-static pressure balance across the nuclear envelope, which responds to dynamic processes in the nucleus such as the active import of proteins, the unfolding of chromatin and DNA replication.

1.1. Pressure balance

We first introduce the concept of pressure balance in phase 2, when the nuclear envelope is formed. This allows us to clearly define the nucleoplasm and cytoplasm, their respective osmotic pressures, and the chromatin pressure.

1.1.1. Pressure balance during phase 2

In phase 2, we assume that the nucleus possesses a fully functional nuclear envelope (NE) maintaining the compartment's identity and integrity. Following previous work [1–3,6], we consider the nuclear volume to be set by the balance of pressures present in the system. In this picture, inward and outward pressures are balanced by the NE where any imbalance results in a change of the nuclear volume via water flux across the nuclear envelope. The pressure balance equation [1] is written as:

$$0 = P_{\mathrm{out}} - P_{\mathrm{in}} \tag{1}$$

where P_{out} denotes the outward pressures increasing nuclear volume and P_{in}, the inward pressures decreasing nuclear volume.

We assume the pressures originating from the nuclear and cytoplasmic compartments to be isotropic, which is supported by the nearly spherical nuclear shapes

at large enough times ($t \geq 15$ min). In a cellular context, non-isotropic pressures can originate from interactions of the NE with the cytoskeleton which, however, has no contribution in our cell-free system. We can also neglect nuclear envelope tension as supported by Supplementary Notes Fig. 8 where we show that the *X. laevis* nucleus behaves like a near-ideal osmometer under hypo-osmotic and control conditions (section 1.4.5). Under strongly hyper-osmotic conditions, the high density of the nucleus leads to deviations from ideal gas law.

The pressures we consider for this model are the osmotic pressure across the nuclear envelope and the outward pressure exerted by chromatin as a confined polymer. In the next sections we will go into the details of how we determine these pressures for our model.

1.1.2 Colloidal osmotic pressure

It has been recently proposed that the osmotic pressures generated by nuclear and cytoplasmic proteins, respectively [1–3,7–9] are the key players contributing to the osmotic balance across the nuclear envelope (see Fig. 4c, d). In the context of osmotic pressure, the nuclear envelope is not simply a semipermeable membrane. The NPCs act as the gateways between the nucleus and cytoplasm through which small molecules can freely diffuse, whereas molecules larger than $\sim 30 - 40$ kDa cannot [10]. As a result, small macromolecules and ions, despite being major osmolytes, do not contribute to the pressure difference (see, however, the discussion of the role of counterions in the main text). Larger proteins need to be specifically transported into the nucleus to establish the nuclear proteome [11–14] (section 1.2.1). Therefore, to estimate the osmotic pressure, we consider the contribution of localized proteins and neglect the effects of ions and other small molecules. In this case, colloid osmotic pressure defines the source of the osmotic pressure, which are mainly proteins [8]. We define ΔP_{osm} as the difference between the outward osmotic pressures exerted by nuclear proteins and the inward osmotic pressure exerted by cytoplasmic proteins, which are determined by the law of van’t Hoff [15]:

$$\Delta P_{osm} = k_B T\left(n_n - n_c\right) \tag{2}$$

where k_B is the Boltzmann constant, T the temperature and $n_{n,c}$ denote the protein concentrations in the nucleus and cytoplasm, respectively. To estimate the protein concentration in the cytoplasm, we use the measured density $\rho_c = 100$ mg mL^{-1}. The cytoplasmic and nucleoplasmic compositions of proteins are characterized by a number average protein mass m_c and m_n , respectively. We also consider that proteins *in vivo* exist as parts of larger complexes, which increases the effective $m_{n,c}$ and consequently reduces the overall protein concentration [8,16]. Using quantitative mass spectrometry data from *X. laevis* oocytes [11], we take $m_c = 155 \pm 1.5$ kDa (weighted average of protein complexes) and calculate the concentration of cytoplasmic protein complexes to be $n_c = \rho_c / m_c \approx 0.65$ mM. Given that macroscopic amounts of extract are present in our system, we assume that the formation of a nucleus does not deplete the extract and thus cytoplasmic quantities such as n_c and m_c remain constant throughout nuclear assembly and growth.

While we can directly use density measurements to calculate protein concentrations for the cytoplasm [17], this is different for the nucleus. Here, we need to consider the contribution of chromatin to i) the density measurements and ii) the nuclear volume available to proteins.

Chromatin has a negligible contribution to the osmotic pressure in the sense of the van't Hoff formula, but it does contribute noticeably to nuclear mass and occupied volume. Therefore, the protein fraction of the nuclear dry mass at a given moment of time can be calculated from the total dry mass of the nucleus as $M_n = M_{tot} - M_{chr}$. To estimate the mass of chromatin M_{chr}, we add up the masses of base pairs and nucleosomes:

$$M_{chr} = N_{bp} m_{bp} + N_{ncl} m_{ncl}$$

$$N_{ncl} = \frac{N_{bp}}{l_{repeat}} \tag{3}$$

$$m_{ncl} = 2\left(m_{h2a} + m_{h2b} + m_{h3} + m_{h4}\right) + m_{h1m}$$

where N_{bp} is the number of base pairs in the genome, m_{bp} denotes the mass of a base pair (0.65 kDa [18]) and N_{ncl} is the number of nucleosomes which can be estimated with the knowledge of the nucleosome repeat length l_{repeat} (200 bp [19]). The mass of a nucleosome m_{ncl} is calculated as the added mass of the histone octamer consisting of two copies of the histones H2A, H2B, H3, and H4 each and one copy of the maternal linker histone H1M. For the *X. laevis* genome of 3.1 Gbp this results in $M_{chr} \approx 6.9$ pg (and double of that after replication). Considering the weighted average of protein complexes in the nucleus leading to $m_n = 131 \pm 1.4$ kDa [11], we can calculate the protein concentration in the nucleus as $n_n = M_n / \left(m_n V_n\right)$ (see, however, discussion of the effect of the excluded volume of chromatin below).

If we now use the above equations and measurements of dry mass to calculate the difference of osmotic pressures in Supplementary Equation (2), we would arrive at $\Delta P_{osm} \approx -330$ Pa (at $t = 60$ min) meaning that the nucleus would rather shrink than grow at this negative pressure difference. By repeating the same calculation of the predicted volumes of the nuclei based on the condition of osmotic pressure balance for all time points, we arrive at the first set of points in Fig. 4c, d (empty squares) that noticeably underestimate the volume. We suggest that this discrepancy can be corrected by considering chromatin pressure.

1.1.3 Chromatin pressure

Next, we consider the outward pressure that chromatin can exert. As chromatin decondenses, it attains its thermodynamically preferred conformation with its

representative size quantified by the radius of gyration R_g [20]. Confinement by the NE, however, results in a nuclear radius smaller than the gyration radius and thus leads to an outward pressure exerted by chromatin on the NE. To quantify the relevance of this effect, we describe chromatin as one self-avoiding polymer chain, which was successful in quantitative description of DNA in various settings [21–23]. For such a polymer confined in a sphere, the chromatin pressure is defined by [1,24]:

$$P_{chr} = \frac{5}{4}\frac{k_B T}{V_n}\left(\frac{R_g}{R_n}\right)^{9/4}$$

$$P_{chr} = \frac{5}{4}\left(\frac{4\pi}{3}\right)^{5/4} k_B T\, l_p^{3/2}\left(\frac{N_{bp}}{\rho_{chr} V_n}\right)^{9/4}. \quad (4)$$

The radius of gyration can be estimated as $R_g \approx l_p^{3/2} L_c^{3/5}$ [25], with the contour length of the polymer L_c and its persistence length l_p . We can estimate L_c with the knowledge of the total number of base pairs in the genome N_{bp} and the linear density of chromatin ρ_{chr} that describes the number of base pairs per unit length via $L_c = N_{bp} / \rho_{chr}$. For the persistence length, we find literature values in the range of $100 - 200$ nm [26], and for the linear density it is $20 - 90$ bp nm^{-1} [19,26,27]. For the first preliminary estimate, we take a linear density value of 40 bp nm^{-1} [19,26] and a persistence length of 150 nm, roughly in the middle of literature range.

If we consider the nucleus at $t = 60$ minutes, one round of DNA replication has been completed doubling the number of base pairs to $N_{bp} = 6.2$ Gbp and resulting in $R_g \approx 600$ µm. The average volume of the nucleus at this time is $V_n \approx 600$ µm^3, corresponding to a nuclear radius $R_n \approx 5$ µm, which is significantly smaller than R_g. Thus, chromatin will contribute to the outward pressure with $P_{chr} \approx 540$ Pa. This value is on the order of magnitude to compensate for the above calculated osmotic pressure difference. We also want to note that the chromatin chain (or multiple chains if we consider the

chromosomes) also exert an osmotic pressure in the sense of the van't Hoff law, but it is clearly negligible compared to the osmotic pressure exerted by protein complexes because the number of chromosomes (a few dozen) is much lower than the number of protein complexes (approx. 10^8). Therefore, the osmotic pressure due to localized protein complexes tries to shrink the nucleus as the cytoplasm is more concentrated than the nucleus, whereas the chromatin pressure expands the nuclear volume.

We have estimated the colloid osmotic pressure and chromatin pressure using the relevant biophysical parameters reported in the literature for *X. laevis, tropicalis* or similar eukaryotic systems. A key consideration is proteins existing as complexes because it reduces their effective concentration, thus lowering their colloid osmotic pressure and making the colloid osmotic pressure difference comparable to the chromatin pressure. By not taking this into consideration or by using parameter values from other systems, the estimated pressures can vary considerably, as observed in e.g. [1].

1.1.4 Comparison of pressure estimates

Here we will compare our pressure estimates with the ones done in [1], where the comparison of chromatin vs osmotic pressures led to almost two orders of magnitude difference, which ultimately led the authors to neglect the contribution of chromatin. The reason for such a difference is twofold and highlights the importance of using accurate, system-specific parameters in model calculations. First, the individual proteins (i.e. polypeptide length) were used to calculate concentrations (not the complexes) and the absolute values of pressures (not their difference) were considered, which together led to a much higher estimate for the osmotic pressure term. Second, chromatin parameters in [1] rely on values for a unicellular system, yeast, which have been reported to have higher compaction ratios compared to somatic cells [28]. We adopted the values reported for somatic systems with lower chromatin compaction thus leading to a higher chromatin pressure in our case. Therefore, our estimates follow the conceptual logic of [1] but use parameters more pertinent to the system considered in this work and, taken together, support the commensurable effects of colloid osmotic pressure *difference* of

cytoplasmic/nuclear compartments and chromatin pressure. To give a clearer overview, in Supplementary Notes Table 1 we depict the relevant parameter values that we used compared to the ones used by [1]. The different parameter values corresponding to the *X. laevis* and human systems, can lead to order of magnitude differences in pressure estimates within the same model.

A recently published advanced computational model of chromatin [29] produced a similar quantitative estimate for chromatin pressure ranging from hundreds of Pa up to kPa depending on the nuclear volume. These considerations show that the value of the chromatin pressure can vary significantly depending on the specific system in question. Therefore, it is important to use accurate, system specific values for the biophysical parameters of the system being considered.

	**Our Study**	**(Deviri and Safran, 2022)**
**Chromatin pressure**	**540 ± 60 Pa (*X. laevis*)**	**30 Pa**
Persistence length	150 nm	180 nm
Compaction ratio	40 bp nm^{-1}	90 bp nm^{-1} (yeast)*
Nuclear volume	570 ± 30 μm^3	1000 μm^3
**Cytoplasmic colloid osmotic pressure**	**1610 ± 50 Pa (complexes)**	**8000 Pa**
**Nuclear colloid osmotic pressure**	**1280 ± 30 Pa (complexes)**	**8000 Pa**
Number avg. weight of cytoplasmic protein complexes	155 ± 1.5 kDa	-
Number avg. weight of nuclear protein complexes	131 ± 1.4 kDa	-
Cytoplasmic protein concentration	0.65 ± 0.02 mM (protein complexes)	4.3 mM
Nuclear protein concentration	0.51 ± 0.02 mM (protein complexes)	4.3 mM

Continuation of Supplementary Notes Table 1		
Cytoplasmic mass density	100 ± 2 mg mL^{-1}	-
Nuclear dry mass	50 ± 2 pg	-

Supplementary Notes Table 1 | Comparison of biophysical quantities. Comparison of our estimates for chromatin and colloid osmotic pressures in the *Xenopus* extract system with the upper bound estimates from Deviri and Safran [1] (bold rows), which use human cell parameters with the exception of the chromatin compaction ratio (marked with a star) where the value was taken from yeast cells. The differences arise from the different parameters that we used (regular rows).

Before moving on, we would like to make some clarifications on the terminology we use in this note:

Osmotic pressure: minimum pressure which needs to be applied to a solution to prevent the inward flow of its pure solvent across a semipermeable membrane, in our case the nuclear envelope.

Colloid osmotic pressure: the osmotic pressure exerted by a solution of macromolecules in contact with a semi-permeable membrane, that allows passive passage of water and ions but not macromolecules [8].

Chromatin pressure: the pressure that arises due to the confinement of the chromatin by the nuclear envelope.

van't Hoff law: formula that describes the osmotic pressure exerted by solutes trapped in a compartment. The concentration of such solutes is in the dilute regime, which makes the osmotic pressure simply be the product of the solute's concentration times the thermal energy of the system.

Dilute regime: describes a solution with low solute concentration. Due to this, interactions of the solute molecules with themselves and other molecules can be neglected. There are cases when solutions with high solute concentration can effectively behave as a dilute

solution (e.g. cells and nuclei).

We can now write the simple pressure balance (which for now ignores the effect of the excluded volume of chromatin, see below) starting from Supplementary Equation (1) and substituting the osmotic pressure from Supplementary Equation (2) and chromatin pressure from Supplementary Equation (4):

$$0 = \frac{M_{\mathrm{n}}}{m_{\mathrm{n}} V_{\mathrm{n}}} - \frac{\rho_{\mathrm{c}}}{m_{\mathrm{c}}} + \frac{5}{4}\left(\frac{4\pi}{3}\right)^{5/4} l_{\mathrm{p}}^{3/2}\left(\frac{N_{\mathrm{bp}}}{\rho_{\mathrm{chr}} V_{\mathrm{n}}}\right)^{9/4}. \tag{5}$$

Supplementary Equation (5) was used for the chromatin-pressure-corrected set of nuclear volumes plotted in Fig. 4c, d of the main text (empty diamonds), which now much better match our experimental data.

In Supplementary Notes Fig. 2 below we show those predictions on the right column for nuclei grown in extract from a *X. laevis* (top row) and *X. tropicalis* (bottom row) chromatin source. On the left column, we show how the predictions would look like assuming that no protein complexes are formed. This would lead to a significantly higher colloid osmotic pressure (see Supplementary Notes Table 2 further below) and even with the addition of chromatin pressure the predictions do not match the experimental data on nuclear volume. This shows the importance of accounting for protein complex formation in the pressure balance across the nuclear envelope.

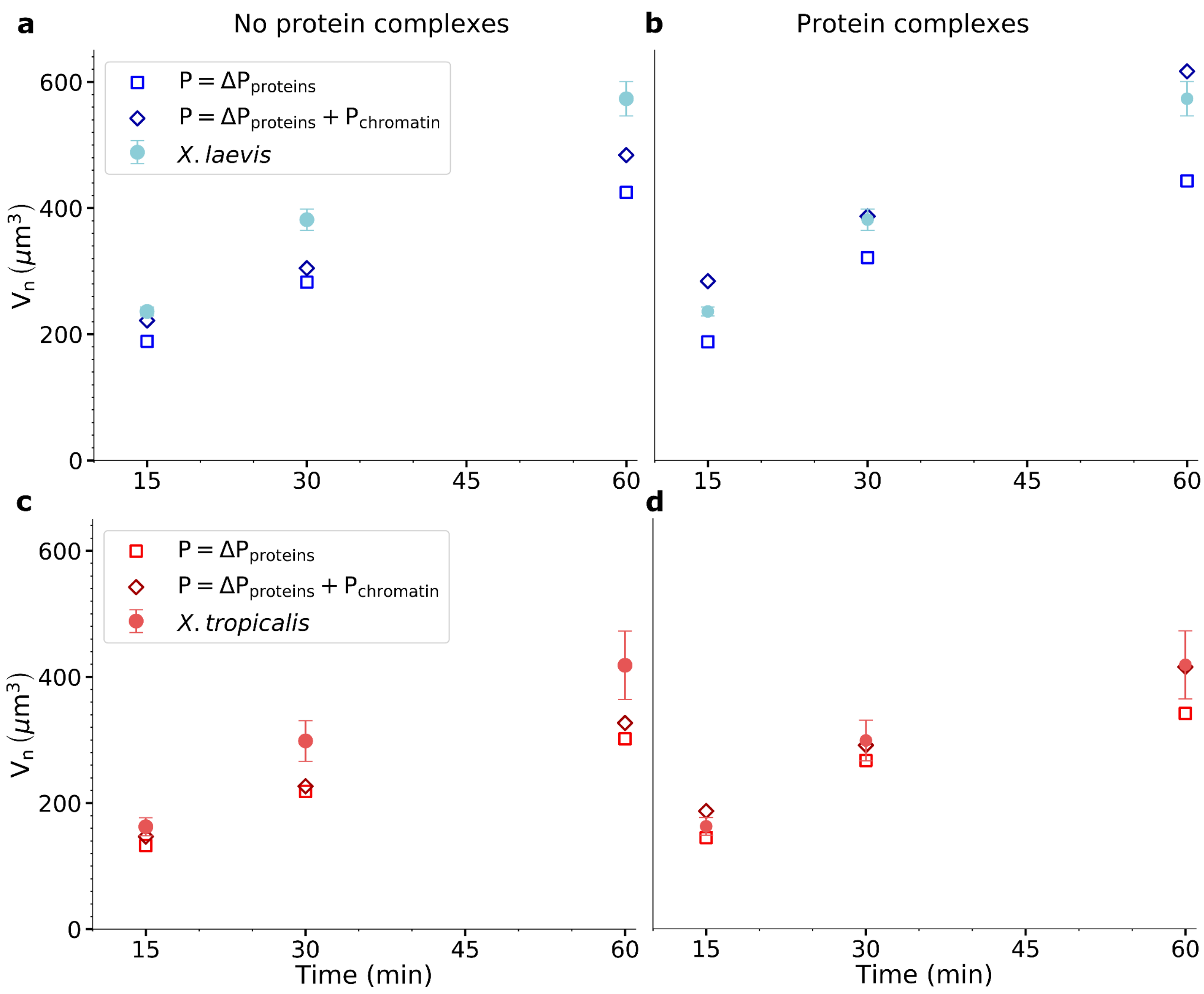

Supplementary Notes Fig. 2 | Comparison of nuclear volume predictions.

(a) Volume predictions assuming no protein complex formation in *X. laevis*.

(b) Volume predictions assuming protein complex formation in *X. laevis*.

(c) Volume predictions assuming no protein complex formation in *X. tropicalis*.

(d) Volume predictions assuming protein complex formation in *X. tropicalis*.

In each graph, solid circles show the experimental data, squares show the volume predictions obtained from a pressure balance considering only the colloid osmotic pressure from proteins and diamonds show the predictions obtained from a balance with colloid osmotic pressure and chromatin pressure, as depicted in Supplementary Equation (5).

In section 1.1.6 below, we will use the pressure balance and experimental measurements of dry mass to calculate the respective chromatin characteristics at different time points. However, before doing that we have a quick look at how the concept of

pressure balance can be useful in phase 1, when there are no well-defined compartments yet.

1.1.5 Excluded volume of chromatin

In phase 1, the nuclear envelope is still forming and thus we can not yet define the nucleus as a separate compartment. Therefore, we formally define nuclear volume as the volume encompassing the chromatin. From the measured dry mass of the nucleus we can calculate the mass of nuclear proteins at $t = 5$ min (we should note that the first time point of $t = 0$ min corresponds to a measurement of sperm chromatin in a buffer solution). Proteins in the available nuclear volume should have equal osmotic pressure to the cytoplasmic proteins and thus equal concentrations:

$$0 = \frac{M_n}{m_n V_n} - \frac{\rho_c}{m_c}.$$

Using m_n (for simplicity we assume the same nuclear identity of proteins at all time points) and the corresponding protein mass M_n, we can calculate the protein number. Using the above equation, we estimate the effective volume available to proteins which is $V_n^{eff} \approx 120$ μm^3 (at $t = 5$ min). This volume is lower than the measured volume $V_n \approx 170$ μm^3 of the nucleus. This suggests that the volume available to proteins is reduced by the space occupied by chromatin. We can then define the effective excluded volume of the chromatin as $V_{chr} = V_n - V_n^{eff}$, resulting in $V_{chr} \approx 50$ μm^3 at $t = 5$ min. This result is close to the estimated minimal value of the physical chromatin volume calculated as the sum of the DNA and nucleosomes' volumes [30] for the replicated *X. laevis* genome ~ 24 μm^3, see Supplementary Notes Table 2 for the exact parameter values used to calculate this estimate.

We assume that for later times the excluded volume of chromatin is not changing till the onset of replication and doubles thereafter. Importantly, the excluded volume of the chromatin will limit the space available for proteins with consequences for protein colloid osmotic pressure in the nucleus:

$$P_{\mathrm{osm,n}} = \frac{M_{\mathrm{n}}}{m_{\mathrm{n}}\left(V_{\mathrm{n}} - V_{\mathrm{chr}}\right)}.$$

1.1.6 Chromatin decondensation and replication

During the course of our observations, chromatin condensation and content are changing. In phase 1, it is the biochemical and steric change leading to its decondensation, while in phase 2, it further relaxes to its equilibrium shape and then proceeds through replication. For simplicity of analytical treatment, we describe chromatin in all stages as a self-avoiding flexible polymer with parameters of number of basepairs N_{bp}, persistence length l_{p}, and linear density ρ_{chr}. The former two can be combined in a parameter $\kappa = l_{\mathrm{p}}^{2/5}\,\rho_{\mathrm{chr}}^{-3/5}$ that determines the gyration radius of the polymer via $R_{\mathrm{g}} = \kappa\, N_{\mathrm{bp}}^{3/5}$ and thus its pressure when in confinement. With this definition and with the excluded volume correction introduced in section 1.1.5, the final pressure balance equation is given by:

$$0 = \frac{M_{\mathrm{n}}}{m_{\mathrm{n}}\left(V_{\mathrm{n}} - V_{\mathrm{chr}}\right)} - \frac{\rho_{\mathrm{c}}}{m_{\mathrm{c}}} + \frac{5}{4}\left(\frac{4\pi}{3}\right)^{5/4} \kappa^{15/4} \left(\frac{N_{\mathrm{bp}}}{V_{\mathrm{n}}}\right)^{9/4}. \qquad (6)$$

Generally, it is difficult to measure chromatin properties directly. We can, however, use our experimental data to determine $\kappa(t)$. In phase 1, we obtain $\kappa(t)$ by simply equating the measured nuclear volume to the volume of the sphere with $R_{\mathrm{n}} = R_{\mathrm{g}}$ (note, however, that it is a strong approximation as shapes of the nuclei at early stages are not spherical yet). In phase 2, we can get the value of $\kappa(t)$ from the pressure balance depicted in Supplementary Equation (6) by inserting the respective measured values for V_{n} and M_{n} and solving for $\kappa(t)$ (note that N_{bp} doubles from around 30 to 60 min due to replication).

Thus, the calculated $\kappa(t)$ plotted as a function of time in Supplementary Notes Fig. 3 reveals the change of two orders of magnitude between phases 1 and 2. It is important to remember, however, that in phase 1, sperm chromatin is initially highly condensed and thus its description via a self-avoiding polymer with the parameter κ during decondensation is at best phenomenological. However, for times ≥ 15 min it should converge to biologically realistic parameters, which it does (as shown by the red-star

symbol in the plot calculated for $l_p = 150$ nm and $\rho_{chr} = 40$ bp/nm). We next approximate $\kappa(t)$ with two fitting functions: i) Michaelis-Menten growth in phase 1, and ii) sigmoidal (exponential) relaxation in phase 2 (see Supplementary Notes Fig. 3 caption for details). We set (without loss of generality) the boundary of the two phases to $t^* = 10$ min (blue symbol), where we also see the onset of nuclear transport in experiments.

So far, we rationalized the relationship between the measured volume of the nucleus, the mass of nuclear proteins, and chromatin at different time points via the pressure balance. We next wanted to understand the time dynamics of nuclear growth.

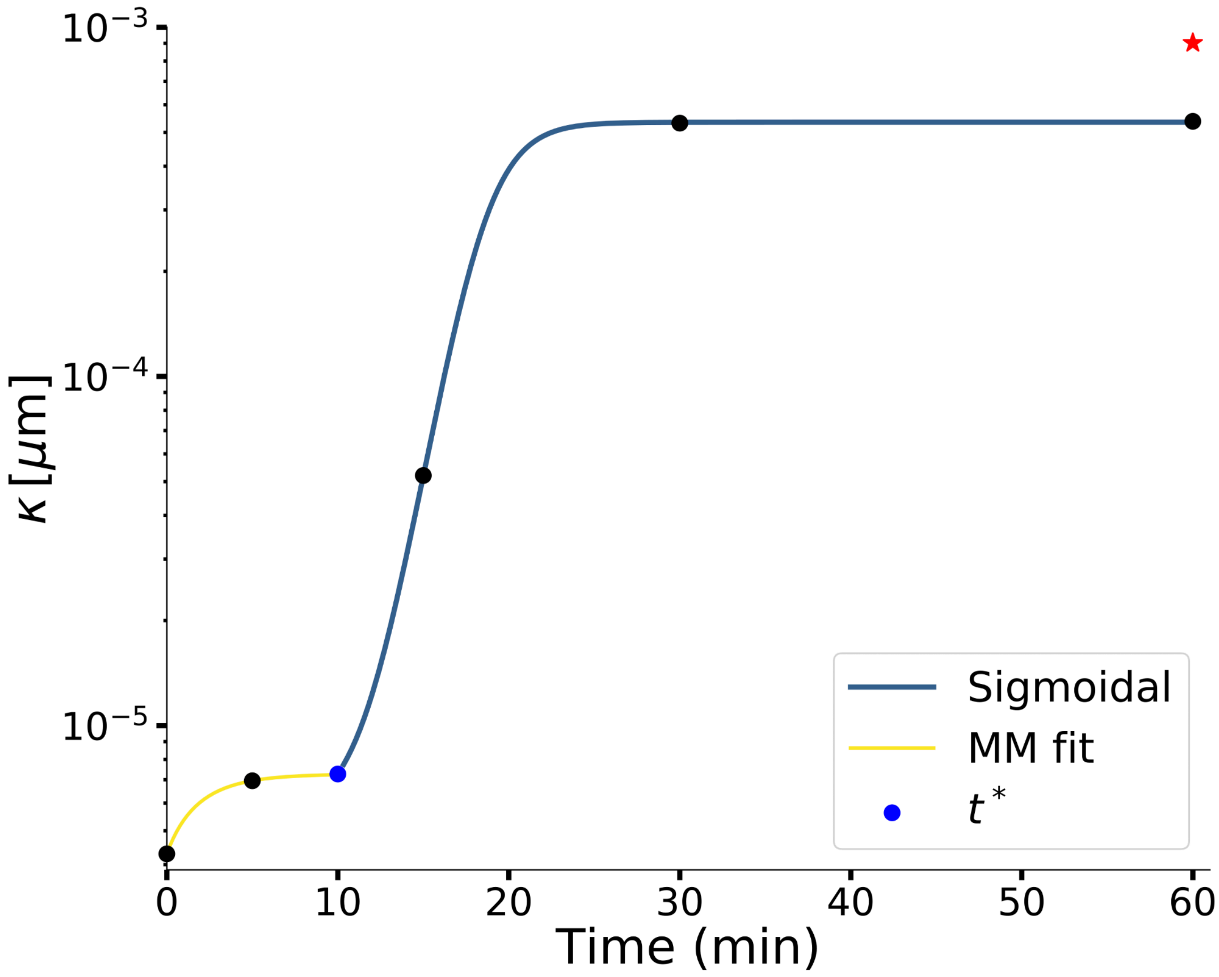

Supplementary Notes Fig. 3 | Quantifying the parameter κ from the pressure balance.

Values of κ are plotted in phase 1 ($t \leq 10$ min) from equating the measured volume of the space occupied by

chromatin to a sphere with a gyration radius as function of κ (see the text), in phase 2 ($t > 10$ min) from the pressure balance depicted in Supplementary Equation (6). The data points are then fitted with a Michaelis-Menten kinetics in phase 1 and simple sigmoidal behavior in phase 2. The fit indicates that once chromatin fully decondenses at around $t = 20$ min, κ and therefore its mechanical properties do no longer change. The star-symbol represents the κ value obtained from literature values of the persistence length $l_{\mathrm{p}} = 150$ nm and compaction ratio $\rho_{\mathrm{chr}} = 40$ bp nm^{-1}. The blue symbol denotes the transition time between the two phases $t^{*} = 10$ min.

1.2 Dynamic model

Our next step is to expand the model enabling us to predict nuclear volume V_{n} and protein content N_{n} as a function of time. In phase 1, the volume of the nucleus just follows the decondensation of chromatin, and the protein mass follows cytoplasmic concentration in the volume of the nucleus reduced by the excluded volume of the chromatin. In phase 2, dynamics are richer. Due to the assumption of instantaneous transport of water, the nuclear volume V_{n} changes in a quasi static manner following the pressure balance depicted in Supplementary Equation (6):

$$\frac{N_{\mathrm{n}}(t)}{\left(V_{\mathrm{n}} - V_{\mathrm{chr}}\right)} - \frac{\rho_{\mathrm{c}}}{m_{\mathrm{c}}} + \frac{5}{4}\left(\frac{4\pi}{3}\right)^{5/4} \kappa(t)^{15/4} \left(\frac{N_{\mathrm{bp}}(t)}{V_{\mathrm{n}}(t)}\right)^{9/4} = 0. \tag{7}$$

During phase 2, active nucleocytoplasmic transport establishes the nuclear proteome. This causes changes in the number of protein complexes in the nucleus $N_{\mathrm{n}} = M_{\mathrm{n}} / m_{\mathrm{n}}$, which in turn affects the osmotic pressure in Supplementary Equation (7). Thus, our model requires a mathematical description of nucleocytoplasmic transport. This transport system has been extensively studied over the years and several models have been proposed, ranging from conceptual to rather detailed [12,31–34].

1.2.1 Kinetic theory of nuclear import

Nuclear pore complexes connect the nuclear and cytoplasmic compartments and only small macromolecules $\leq 30 - 40$ kDa [10] are able to freely translocate through them. Larger proteins need to be selectively transported through the pores by nuclear transport

receptors, such as importins. Importins bind to cargo proteins via their nuclear localization signal (NLS). The resulting cargo-importin complex is able to passively shuttle between the nuclear and cytoplasmic compartments, enabled by specific interactions of the importin with NPCs. Accumulation of cargo in the nucleus is achieved by the asymmetric distribution of the GTPase Ran: the nucleus is enriched with GTP-bound Ran (RanGTP), while the cytoplasm is enriched with GDP-bound Ran (RanGDP). RanGTP has a high binding affinity to importins, which along with its abundance in the nucleus leads to the formation of RanGTP-importin complexes, thus dissolving the cargo-importin complexes and delivering cargo in the nucleus [33,35]. This is how specific proteins accumulate in the nucleus. The asymmetric distribution of RanGTP is achieved by a dedicated molecular machinery. The GTPase-activating protein RanGAP, which is localized to the cytoplasmic side of the NE, hydrolyses the RanGTP of outgoing RanGTP-importin complexes, thus dissolving the complex and producing free RanGDP and importin as a result. Another transport receptor NTF2 binds to RanGDP in the cytoplasm, forming a complex that shuttles Ran back to the nucleus. Once the complex enters the nucleus, the guanine-nucleotide exchange factor RCC1 (or RanGEF), which is localized to chromatin [36,37], converts RanGDP into RanGTP. This dissolves the complex and liberates RanGTP in the nucleus. In summary, the accumulation of specific proteins in the nucleus is achieved by the action of nuclear transport receptors such as importin and the asymmetric distribution of RanGDP/RanGTP, which is maintained by the localization of RanGAP to the outside and RanGEF to the inside of the nucleus. Since this process consumes energy by hydrolysis of RanGTP to RanGDP, it is also referred to as a directed active transport. Throughout the years, multiple biological and biophysical models of varying complexity have been proposed by various authors [31,33,38–42]. For the reconstituted nuclei of *X. laevis*, Kopito and Elbaum [31] put forward an experimental demonstration and proposed a simple kinetic theory of this process (see also [12,33]). Here, we build up on the model of [12] by explicitly linking the transport equation to the chromatin-dependent RanGEF concentration and consider it in the context of dynamically expanding nuclei as governed by the pressure

balance. One of the findings of [32] is that accumulation of cargo in the nucleus of fixed size can be described by an effective first order kinetics equation. Concentration of the NLS cargos follows a simple differential equation:

$$\frac{dn_{\mathrm{n}}}{dt} = k_{\mathrm{in}} n_{\mathrm{c}} - k_{\mathrm{out}} n_{\mathrm{n}}, \tag{8}$$

where k_{in}, k_{out} respectively denote the effective forward and backward rates of the first-order kinetics and $n_{\mathrm{c,n}}$ are the concentration of NLS cargo in the two compartments. For clarity of argument, we assume that $n_{\mathrm{c,n}}$ represents an effective single species NLS protein. We argue that these effective kinetics, driven by the Ran gradient, will depend on RCC1 (RanGEF) concentration, which in turn correlates with chromatin content [43]. To show how the effective dynamics depend on RanGEF concentration, we reproduce the core reactions of the import cycle reported in [12]:

$$[\mathrm{N}]_{\mathrm{c}} + [\mathrm{R'}]_{\mathrm{c}} \underset{k_1^-}{\overset{k_1^+}{\rightleftharpoons}} [\mathrm{NR'}]_{\mathrm{c}} \tag{9}$$

$$[\mathrm{TR}]_{\mathrm{c}} \underset{k_2^-}{\overset{k_2^+}{\rightleftharpoons}} [\mathrm{T}]_{\mathrm{c}} + [\mathrm{R'}]_{\mathrm{c}} \tag{10}$$

$$[\mathrm{TR}]_{\mathrm{n}} \underset{k_3^-}{\overset{k_3^+}{\rightleftharpoons}} [\mathrm{TR}]_{\mathrm{c}} \tag{11}$$

$$[\mathrm{T}]_{\mathrm{n}} + [\mathrm{R}]_{\mathrm{n}} \underset{k_4^-}{\overset{k_4^+}{\rightleftharpoons}} [\mathrm{TR}]_{\mathrm{n}} \tag{12}$$

$$[\mathrm{NR'}]_{\mathrm{n}} \underset{k_5^-}{\overset{k_5^+}{\rightleftharpoons}} [\mathrm{N}]_{\mathrm{n}} + [\mathrm{R}]_{\mathrm{n}} \tag{13}$$

$$[\mathrm{NR'}]_{\mathrm{c}} \underset{k_6^-}{\overset{k_6^+}{\rightleftharpoons}} [\mathrm{NR'}]_{\mathrm{n}} \tag{14}$$

$$[\mathrm{TC}]_{\mathrm{n}} \underset{k_7^-}{\overset{k_7^+}{\rightleftharpoons}} [\mathrm{T}]_{\mathrm{n}} + [\mathrm{C}]_{\mathrm{n}} \tag{15}$$

$$[\mathrm{T}]_{\mathrm{c}} \underset{k_8^-}{\overset{k_8^+}{\rightleftharpoons}} [\mathrm{T}]_{\mathrm{n}} \tag{16}$$

$$[\mathrm{TC}]_c \underset{k_9^-}{\overset{k_9^+}{\rightleftharpoons}} [\mathrm{TN}]_n \tag{17}$$

$$[\mathrm{T}]_c + [\mathrm{C}]_c \underset{k_{10}^-}{\overset{k_{10}^+}{\rightleftharpoons}} [\mathrm{TC}]_c \tag{18}$$

$$[\mathrm{N}]_n \underset{k_{11}^-}{\overset{k_{11}^+}{\rightleftharpoons}} [\mathrm{N}]_c \tag{19}$$

where the subindexes n, c denote nuclear and cytoplasmic quantities, respectively. The abbreviations we used stand for N : NTF2, R : RanGTP, R' : RanGDP, T : Importin, C : Cargo Protein. We can classify these reactions in three types: binding/ unbinding in the cytoplasm (Supplementary Equations (9), (10), (18)), binding/ unbinding in the nucleus (Supplementary Equations (12), (13), (15)) and passive diffusion through the NPCs (Supplementary Equations (11), (14), (16), (17), (19)). For this set of reactions, the corresponding set of standard chemical rate equations can be written down, for which we use the same equations as in the original work [12].

We note that Supplementary Equation (13) is where the nuclear NTF2-RanGDP is dissociated and RanGEF exchanges RanGDP for RanGTP. Therefore, the influence of RanGEF is captured in the forward rate k_5^+ leading to our assumption that $k_5^+ \propto [\mathrm{RanGEF}]_n$, which is not considered in the original work [12]. In a similar fashion, we can make the assumption $k_2^+ \propto [\mathrm{RanGAP}]_c$.

We can coarse grain the detailed transport model proposed in [12] to the effective first order kinetics depicted in Supplementary Equation (8) by considering the steady state of the full system, Supplementary Equations (9)-(19) and comparing it to the steady state of Supplementary Equation (8). After considerable algebra, we derived expressions for k_{in} and k_{out} in terms of the parameters of the detailed transport model of [12]:

$$k_{\mathrm{in}} = k_7^+ \frac{k_9^+}{k_9^-} T_0 \frac{1}{\kappa_c^{TC} + [C]_c} \tag{20}$$

$$k_{\mathrm{out}} = P\, k_7^- \frac{\kappa_c^{TR}}{\kappa_n^{TR}\kappa_n^{NR'}} \frac{[N]_c [T]_c}{N_0} \left(\kappa_c^{NR'} + [R']_c \right) \tag{21}$$

where:

$$P = \frac{k_3^- k_6^- k_{11}^-}{k_3^+ k_6^+ k_{11}^+}, \quad \kappa_n^{TR} = \frac{k_4^+}{k_4^-}, \quad \kappa_c^{NR'} = \frac{k_1^-}{k_1^+}, \quad \kappa_n^{NR'} = \frac{k_5^+}{k_5^-}, \quad \kappa_c^{TR} = \frac{k_2^-}{k_2^+}.$$

We can see that k_{in} only depends on cytoplasmic quantities and can be indeed assumed to be constant in our model under the assumption that nuclear assembly does not affect the cytoplasmic composition. Importantly, for k_{out} we get:

$$k_{out} \propto \frac{\kappa_c^{TR}}{\kappa_n^{NR'}} \propto \frac{1}{[\mathrm{RanGEF}]_n [\mathrm{RanGAP}]_c} \propto \frac{V_n}{N_{GEF}}. \tag{22}$$

Therefore, the dependency of k_{out} on RanGEF concentration brings in a dependency of import efficiency on the nuclear volume V_n, and the amount of RanGEF in the nucleus N_{GEF}. We assume that the amount of RanGEF in the nucleus is correlated with the amount of chromatin and would increase with replication of chromatin. This means that replication will decrease the value of k_{out} because the value of N_{GEF} increases. As we do not know the exact value of N_{GEF} and how it changes during replication, we assume that replication reduces the value of k_{out} by a phenomenological factor $\nu \leq 1$.

The model presented in [12] can be considered as a general model for the import of proteins into the nucleus, which takes into account the shuttling of cargo proteins by importin and the asymmetric distribution of RanGTP across the nuclear envelope. However, depending on the specific system in question, other effects could be included in the model. For instance, the kinetic model recently used by [34] (as well as our model), is essentially based on the model of the Elbaum group [31,32]. While their model includes the perspective of mechanosensitivity of the transport, our model further describes the dependence of the GTP-gradient on the RanGEF amount (which we assume to be linked to the amount of chromatin). Therefore our model is fully consistent in the part of the kinetic model of transport but additionally includes the dependence on RCC1 concentration, which, in [34] is absorbed in a single parameter γ (see their Supplemental

Information). Therefore, our transport model is more comprehensive for our experimental system.

A possible source of concern in our modeling is that we do not explicitly account for the export of proteins from the nucleus to the cytoplasm. While there is a coupling effect of nuclear import/export, this is currently absorbed in the phenomenological k_{in} and k_{out} parameters of our model. This is a reasonable simplification as there is no transcription or ribosome biogenesis in the *Xenopus* egg extract system. Therefore, the main job of export is to keep cytoplasmic proteins out of the nucleus. Furthermore, we assumed cytoplasmic mass density to be constant and therefore, the cytoplasmic protein concentration is not affected by transport in the extract system, where nuclei are formed in a virtually unlimited pool of cytoplasm. Consistently, it has been shown previously that in *Xenopus* oocytes and eggs only a small fraction of proteins responds to export inhibition [11,44]. If we were to extend to a model of a nucleus nested in a cell, where cytoplasmic components can be limiting, then it would make sense to explicitly consider the identities of cytoplasmic proteins (as we do now for nuclear proteins) and add more transport equations that represent export and other effects.

After using the model [12,31] to establish the dependence of the effective first order kinetics of transport on the concentration of RanGEF for the steady-state volume, we are ready to push it to the dynamical regime where volume changes over time.

1.2.2 Time-dependent nuclear volume dynamics

In our experimental setup, the nuclear volume $V_{\mathrm{n}}(t)$ is a variable while the cytoplasmic concentration of any protein (including our effective nuclear protein) is constant, i.e. $n_{\mathrm{c}} = \mathrm{const}$ [45]. Thus, for the time derivative of the effective nuclear protein concentration we have:

$$\frac{dn_{\mathrm{n}}}{dt} = \frac{1}{V_{\mathrm{n}}}\frac{dN_{\mathrm{n}}}{dt} - \frac{N_{\mathrm{n}}}{V_{\mathrm{n}}^2}\frac{dV_{\mathrm{n}}}{dt}. \tag{23}$$

Concentration of nuclear proteins changes due to the number of protein complexes N_{n} shuttling through the NE, but also due to the changes in its volume V_{n}. Thus, using the first order kinetics depicted in Supplementary Equation (8) and noting that $dV_{\mathrm{n}} / dt = 0$ because of the instantaneous water equilibration [3–5], we can write the time derivative for the number of nuclear proteins:

$$\frac{dN_{\mathrm{n}}}{dt} = k_{\mathrm{in}} V_{\mathrm{n}} n_{\mathrm{c}} - k_{\mathrm{out}} N_{\mathrm{n}}. \tag{24}$$

With all the considerations explained above, we arrive at the final set of equations for our model, where the nuclear volume changes in a quasi steady-state fashion. In particular, we include the RanGEF dependence of k_{out} by means of the substitution $k_{\mathrm{out}} \rightarrow k'_{\mathrm{out}} V_{\mathrm{n}}(t) / \nu(t)$, where $k'_{\mathrm{out}} = \mathrm{const}$ and $\nu(t)$ reflects the maintenance of import by chromatin content:

$$\frac{dN_{\mathrm{n}}}{dt} = k_{\mathrm{in}} V_{\mathrm{n}}(t) \frac{\rho_{\mathrm{c}}}{m_{\mathrm{c}}} - \frac{k'_{\mathrm{out}}}{\nu(t)} V_{\mathrm{n}}(t)\, N_{\mathrm{n}}(t) \tag{25}$$

$$0 = \frac{N_{\mathrm{n}}(t)}{\left(V_{\mathrm{n}}(t) - V_{\mathrm{chr}}\right)} - \frac{\rho_{\mathrm{c}}}{m_{\mathrm{c}}} + \frac{5}{4}\left(\frac{4\pi}{3}\right)^{5/4} \kappa(t)^{15/4} \left(\frac{N_{\mathrm{bp}}(t)}{V_{\mathrm{n}}(t)}\right)^{9/4}. \tag{26}$$

Taken together, the number of nuclear proteins changes in time following Supplementary Equation (25) describing nuclear import, whereas the volume responds as determined by solving Supplementary Equation (26) of pressure balance for V_{n}. Note that $N_{\mathrm{bp}}(t)$ and the factor $\upsilon(t)$ are time-dependent because we are incorporating chromatin replication, and $\kappa(t)$ characterizes chromatin decondensation (section 1.1.6 above and Supplementary Notes Fig. 3, we should note that our data suggests that replication does not affect κ of the chromatin). The effect of replication is captured by doubling the value of N_{bp} in a linear fashion:

$$N_{\mathrm{bp}}(t) = N_{\mathrm{bp}}\left(1 + \frac{\Delta N}{\Delta t} \cdot t\right), \quad t_{\mathrm{r,i}} \le t < t_{\mathrm{r,f}}$$

$$N_{\mathrm{bp}}(t) = 2N_{\mathrm{bp}}, \qquad t \ge t_{\mathrm{r,f}} \tag{27}$$

where $t_{r,i}$ denotes the time at which DNA replication starts, $t_{r,f}$ the time at which DNA replication ends, $\Delta N = 1$ and $\Delta t = t_{r,f} - t_{r,i}$. The value of $\nu(t)$ changes similarly:

$$\nu(t) = \nu_i + \frac{\Delta\nu}{\Delta t}\cdot t, \quad t_{r,i} \leq t < t_{r,f}$$

$$\nu(t) = \nu_{r,f}, \qquad t \geq t_{r,f} \tag{28}$$

where $\nu_i = 1$ and the value of ν_f is considered to be a model parameter, which leaves $\Delta\nu = \nu_f - 1$. As we will proceed to show next, the model relies on a very limited number of free parameters with most of them taken from literature or directly recovered from our experiments.

1.3 Parameters

We use the control conditions to set most of the model parameters. We then show how selected parameters need to be adjusted for the respective experimental perturbations. The final time point of our experimental measurements is minutes after one round of DNA replication. Therefore, we assume the final experimental time point to reflect the steady-state of the system although nuclei keep growing [46]. This means that we can use our experimental values to set the values of the steady-state quantities N_n^{SS} and V_n^{SS}. The mean volume measured at 60 min sets V_n^{SS} and N_n^{SS} can be obtained with the ratio of the mean protein mass M_n at 60 min and the number average protein mass in the nucleus m_n. With this knowledge, we can estimate the value of the ratio k'_{out} / k_{in} by considering the steady-state of the transport kinetics, Supplementary Equation (25):

$$\frac{k'_{out}}{k_{in}} = \nu_f \frac{\rho_c}{m_c N_n^{SS}}.$$

We take k_{in} as a model parameter and can determine the value of k'_{out} with the equation above. Taken together, this leaves us with just three free model parameters: k_{in}, $\kappa(t)$ and ν_f. All other parameters are either known from literature or directly calculated from

measurements in this work. In Supplementary Notes Table 2 we provide an overview of the biophysical quantities relevant to our *Xenopus* extract system. The model parameters that are varied for the theoretical simulations of the different experimental conditions are listed in Supplementary Notes Table 3.

Quantity	Value	Unit	Source
**Cytoplasmic density**	100 ± 2	**mg mL^{-1}**	**Our measurements**
Nuclear density (60 min)	91.4 ± 0.8	mg mL^{-1}	Our measurements
Nuclear volume (60 min)	570 ± 30	µm^3	Our measurements
Cytoplasmic osmolality	307 ± 2	mOsm kg^{-1}	Our measurements
**Total nuclear dry mass (60 min)**	51 ± 2	**pg**	**Derived from measurements**
Protein concentration in cytoplasm	0.65 ± 0.02	mM	Derived from measurements
Protein concentration in nucleus	0.51 ± 0.02	mM	Derived from measurements
Cytoplasmic colloid osmotic pressure (complexes)	1.61 ± 0.05	kPa	Derived from measurements
Cytoplasmic colloid osmotic pressure (no complexes)	6.4 ± 0.2	kPa	Derived from measurements
Nuclear colloid osmotic pressure (complexes, Xl)	1.28 ± 0.04	kPa	Derived from measurements
Nuclear colloid osmotic pressure (no complexes, Xl)	3.57 ± 0.10	kPa	Derived from measurements
Nuclear colloid osmotic pressure (complexes, Xt)	1.38 ± 0.07	kPa	Derived from measurements
Nuclear colloid osmotic pressure (no complexes, Xt)	3.9 ± 0.2	kPa	Derived from measurements
Chromatin pressure (Xl)	0.54 ± 0.06	kPa	Derived from measurements

**Continuation of Supplementary Notes Table 2**			
Chromatin pressure (Xt)	0.28 ± 0.07	kPa	Derived from measurements
Number of nuclear proteins (complexes, Xl)	$(1.67 \pm 0.09) \cdot 10^{8}$	-	Derived from measurements
Number of nuclear proteins (complexes, Xt)	$(1.17 \pm 0.14) \cdot 10^{8}$	-	Derived from measurements
**Number avg. weight of nuclear proteins (complexes)**	131.1 ± 1.4	**kDa**	[11]
**Number avg. weight of nuclear proteins (no complexes)**	39.0 ± 0.3	**kDa**	[11]
**Number avg. weight of cytoplasmic proteins (complexes)**	155.8 ± 1.5	**kDa**	[11]
**Number avg. weight of cytoplasmic proteins (no complexes)**	47.1 ± 0.4	**kDa**	[11]
**Genome length (Xl)**	3.1	**Gbp**	[47]
**Genome length (Xt)**	1.7	**Gbp**	[47]
**Nucleosome repeat length**	200	**bp**	[19]
**Avg. mass of basepair**	0.65	**kDa**	[18]
**Mass of H2.A (Xl)**	14	**kDa**	[48]
**Mass of H2.B (Xl)**	14	**kDa**	[49]
**Mass of H3 (Xl)**	15	**kDa**	[50]
**Mass of H4 (Xl)**	11	**kDa**	[51]
**Mass of B4 linker histone (Xl)**	29	**kDa**	[52]
Chromatin persistence length	$100 - 200$	nm	[26]
Chromatin linear density	$20 - 90$	bp nm^{-1}	[19,26,27]
Linear density of DNA double helix	2.9	bp nm^{-1}	[19]

Continuation of Supplementary Notes Table 2			
Thickness of DNA double helix	2	nm	[53]
Radius of histone octamer	5.5	nm	[53]
Length of histone octamer	6.6	nm	[53]

Supplementary Notes Table 2 | Biophysical quantities relevant to our cell nucleus model in *Xenopus* egg extract.

Most values correspond to fully grown nuclei from *X. laevis* sperm chromatin. The quantities that are used directly in the model equations are marked in bold. Different colors denote the origin of the values: green for quantities that are measured in our experiments, blue for quantities that are derived from our measurements and/or the literature, black for quantities that are taken from the literature. Additional information is provided in parenthesis: the time at which the quantity was measured, the frog species used as a chromatin source (Xl for *X. laevis* and Xt for *X. tropicalis*) and the assumed configuration of proteins (as complexes or single proteins).

	κ_{60} $[10^{-4}\mu m]$	ν_f	k_{in} $[10^{-3}s^{-1}]$	k'_{out} $[10^{-6}\ s^{-1}\ m^{-3}]$	V_{chr} $[\mu m^3]$	m_n [kDa]	m_c [kDa]	ρ_c $[mg\ mL^{-1}]$	N_{bp} [Gbp]
**Ctrl**	5.1 ± 0.6	1.15	1.43 ± 0.07	3.18 ± 0.19	48 ± 4	131	155	100	3.1
**-Imp**	2.5 ± 0.5	1.15	1.37 ± 0.07	5.33 ± 0.17	40 ± 9	131	155	100	3.1
**Xt**	6.5 ± 1.4	1.15	1.5	4.20 ± 0.17	16 ± 6	131	155	100	1.7
**-Rep**	2.5 ± 0.5	1	1.07 ± 0.03	6 ± 1	48 ± 3	150	155	100	3.1
**HS**	6.1 ± 0.8	1.15	2.0 ± 0.4	3.2 ± 0.4	65 ± 20	125	120	53.7	3.1

Supplementary Notes Table 3 | Simulation parameter values used for different experiments.

Free, adjustable parameters in red; parameters estimated from measurements in blue; parameters directly taken from either our own measurements or literature in black. Each row corresponds to: nuclei grown under

control conditions (Ctrl), under import inhibition (-Imp.), with *Xt* sperm chromatin (Xt), under replication inhibition (-Rep.) and finally, in high-speed extract (HS). See section 1.5.4 for details on how the errors were obtained.

1.4 Perturbation experiments

To gain a better understanding of the mechanistic processes determining nuclear volume and density, we perturbed our experimental system in various ways. Our goal in this section is not only to fit our theory to the experimental data for the various perturbations through parameter fine tuning, but we also aim to make the parameter changes consistent with the respective perturbation.

1.4.1 Nuclear import inhibition

We start with describing the nuclear import inhibition experiments. To perturb nuclear import, an inhibitor cocktail of Ivermectin and Pitstop-2 was used. Ivermectin is a broad-spectrum Importin α/β inhibitor [54] and Pitstop-2 is a Clathrin inhibitor that compromises nuclear permeability by modifying NPC structure [55]. Upon import inhibition, the accumulation of NLS-GFP was greatly reduced (Fig. 3a, b). Further, there was a reduction of nuclear volume and dry mass in comparison to control nuclei (Fig. 3c, e). Importantly, the density of reconstituted nuclei did not go below the cytoplasmic density of 100 ± 2 mg mL^{-1} (Fig. 3d).

To reproduce these conditions in our model, we reduce the influx and outflux rates k_{in} and k_{out}. In addition, the DNA fluorescence measurements suggest that our import perturbation also inhibits DNA replication and thus, we keep N_{bp}constant and set ν_{f} to 0. The inhibition of import can be expected to change the identity of the nuclear proteome, which is represented by the number average molecular weight of protein complexes m_{n}. If import is inhibited, we expect the proteome of the perturbed nuclei to remain more similar to the cytoplasmic proteome. Therefore, we can assume m_{n} to take values higher than in control nuclei and closer to m_{c}. An important experimental observation in import-inhibited

nuclei, is that their density is equal to that of the cytoplasm. Together this would lead to very similar osmotic pressures in the cytoplasm and nucleus. This suggests that chromatin pressure is negligible w.r.t. protein colloid osmotic pressure, which is reflected in our model by the fitted κ values which are lower than in the control (Supplementary Notes Table 3).

Taken together, with a negligible chromatin pressure and m_n being similar to m_c, our theory is able to reproduce a smaller nucleus with a density almost identical to the cytoplasmic density (Supplementary Notes Fig. 4). The import inhibition treatment has a combined effect on transport and replication. We can, however, next look at the effects of replication inhibition directly.

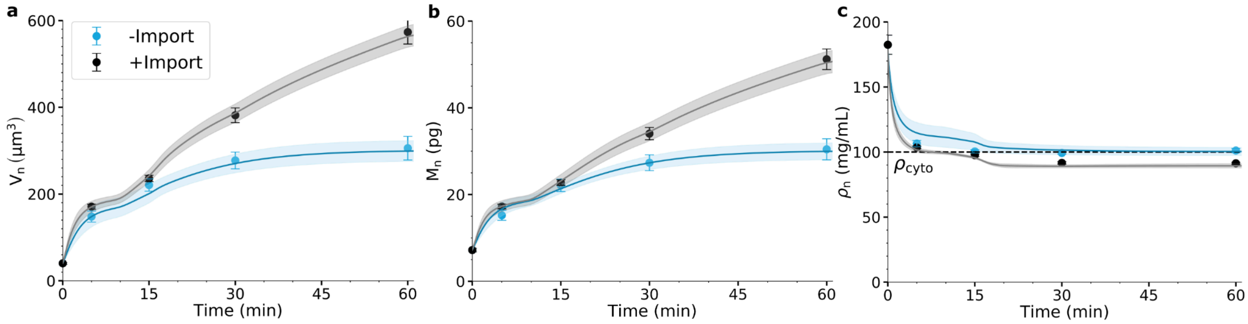

Supplementary Notes Fig. 4 | Simulation of the dynamic model to import inhibition experiments.

(a) Simulation of nuclear volume as a function of time (also depicted in Fig. 3c).

(b) Simulation of nuclear dry mass as a function of time (also depicted in Fig. 3e).

(c) Simulation of nuclear density as a function of time (also depicted in Fig. 3d). Dashed line shows the cytoplasmic density ($\rho_{cyto} = 100$ mg mL^{-1}).

Data related to control conditions (+Import) is depicted in black and data related to import inhibition experiments, in blue (-Import). Dots represent experimental data and solid lines are the simulation results. Error bars and shaded areas are respectively showing the SEM from experiments and simulations. Respective parameters can be found in Supplementary Notes Table 3 (-Imp. condition).

1.4.2 Replication inhibition

To perturb replication, we used the DNA polymerase α inhibitor Aphidicolin [56]. Aphidicolin treated nuclei were significantly smaller than control nuclei and had a lower dry mass but

similar density (Supplementary Fig. 4f-h). In addition, NLS-GFP accumulation was reduced in these nuclei (Supplementary Fig. 4e).

In this condition, we keep N_{bp} constant throughout the whole simulation as well as ν_f with a value of 1, in order to mimic replication inhibition. The reduced NLS-GFP accumulation observed in this perturbation indicates that transport is also affected. This not only suggests that chromatin and transport are linked, but also allows us to change the rates k_{in} and k'_{out} to adjust the steady-state dry mass. As we already argued in the case of import inhibition, we can change the value of m_n under the assumption that the nuclear proteome is changed due to the perturbation. Finally, given that this perturbation acts on DNA, it is reasonable to expect that chromatin properties are also going to change [56], which is seen in the lower fitted value of κ. Taken together, also in this condition we can recapitulate experimental observations (Supplementary Notes Fig. 5).

Since transport is affected by our replication inhibition experiments, we also performed a numerical experiment that represents an "ideal" inhibition of DNA replication, which is depicted as the blue curves in Supplementary Notes Fig. 5. In this simulation, the evolution of nuclear volume, dry mass and dry mass density is identical to control before the start of DNA replication at $t = 30$ min, which is achieved by using the same values for the fit parameters as in the control simulations. The only difference is that the number of basepairs N_{bp} is kept constant as well as ν_f with a value of 1. As a result, after $t = 30$ min there is no increase in chromatin pressure due to a higher N_{bp} and no enhanced import of proteins due to ν_f being constant and unaffected by replication. This leads to nuclear volume, dry mass and dry mass density stalling after $t = 30$ min. Since DNA replication does not affect nuclear mass density in control experiments, its evolution in the ideal replication inhibition is identical to control.

The replication inhibition interfered with chromatin via drug treatments. We can, however, change the chromatin content in a more natural way, by using sperm chromatin of a different frog species.

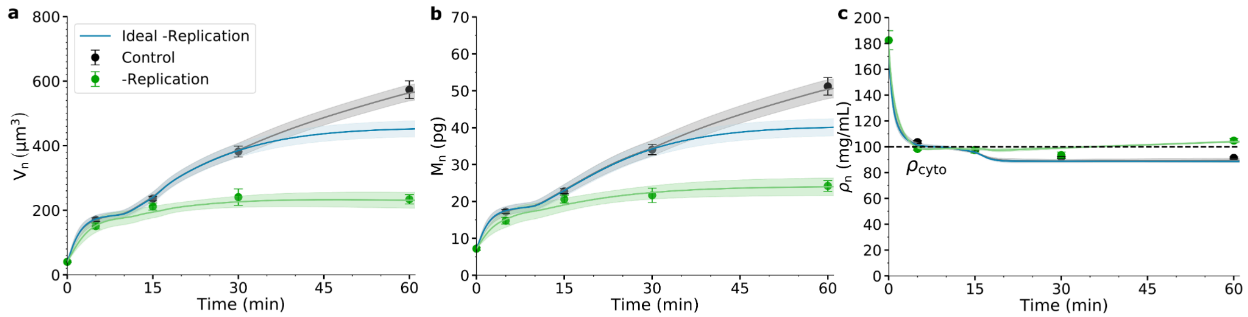

Supplementary Notes Fig. 5 | Simulation of the dynamic model to replication inhibition experiments.

(a) Simulation of nuclear volume as a function of time (also depicted in Supplementary Fig. 4f).

(b) Simulation of nuclear dry mass as a function of time (also depicted in Supplementary Fig. 4g).

(c) Simulation of nuclear density as a function of time (also depicted in Supplementary Fig. 4h). Dashed line shows the cytoplasmic density ($\rho_{cyto} = 100$ mg mL^{-1}).

Data related to control conditions (Control) is depicted in black and data related to import inhibition experiments, in green (-Replication). In addition, depicted in blue is a simulation of an ideal replication inhibition (Ideal -Replication), which is identical to the simulation of the control experiment but has no increase in the number of basepairs after t = 30 min and consequently, no enhancement of protein import due to replication. Dots represent experimental data and solid lines are the simulation results. Error bars and shaded areas are respectively showing the SEM from experiments and simulations. Respective parameters can be found in Supplementary Notes Table 3 (-Rep. condition).

1.4.3 Nuclei assembled around *X. tropicalis* sperm chromatin

To evaluate the effect of chromatin content on nuclear volume, dry mass and density, *X. tropicalis* (*Xt*) sperm, which has a genome half-size of *X. laevis* (*Xl*), was used as a chromatin source in the nuclear assembly reaction. Reconstituted *Xt* nuclei were significantly smaller than control *Xl* nuclei (Fig. 4c, d). Additionally, these nuclei had a lower dry mass but the same density as control nuclei (Fig. 4g, e).

One obvious change is to adjust the value of N_{bp} for the genome of *Xt*. A significantly smaller *Xt* genome results in a lower chromatin pressure, which is essential to

reproduce the smaller volumes observed in *Xt* nuclei (Fig. 4c, d). Naturally, the value we obtain for V_{chr} is lower compared to control. The fitted κ value for *Xt* is higher compared to control (Supplementary Notes Table 3), but considering their errors, we can attribute this difference to natural variations.

Given that the cytoplasmic composition remains the same, we leave the value of k_{in} unchanged compared to control (Supplementary Equation (20)) and only adjust the value of k'_{out} which depends on chromatin content, in particular the amount of RanGEF which we can expect to be lower compared to *Xl* (Fig. 4f). A lower amount of RanGEF is expected to increase the value of k'_{out} (Supplementary Equation (22)), which leads to a lower value of steady-state nuclear dry mass. Taken together, this choice of parameters leads to the theoretical nuclear density of *Xt* being almost identical to *Xl* and a good agreement between experiment and theory (Supplementary Notes Fig. 6).

Chromatin content can indeed affect both nuclear volume and dry mass, however, nuclear mass density was still lower than cytoplasmic mass density. We can grow nuclei in a more diluted cytoplasm and check whether it has an effect on the nuclear to cytoplasmic density ratio.

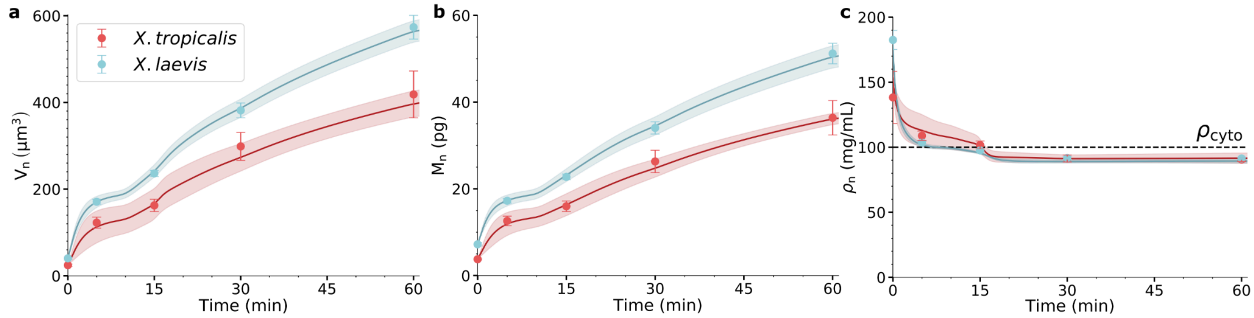

Supplementary Notes Fig. 6 | Simulation of the dynamic model to experiments of nuclei grown from *X. tropicalis*.

(a) Simulation of nuclear volume as a function of time (also depicted in Fig. 4c, d).

(b) Simulation of nuclear dry mass as a function of time (also depicted in Fig. 4g).

(c) Simulation of nuclear density as a function of time (also depicted in Fig. 4e). Dashed line shows the cytoplasmic density ($\rho_{\text{cyto}} = 100$ mg mL^{-1}).

Data related to control conditions (*X. laevis*) is depicted in blue and data related to experiments of nuclei grown from *X. tropicalis* sperm chromatin (*X. tropicalis*), in red. Dots represent experimental data and solid lines are the simulation results. Error bars and shaded areas are respectively showing the SEM from experiments and simulations. Respective parameters can be found in Supplementary Notes Table 3 (Trop. condition).

1.4.4 Growth in high-speed extract

To evaluate the importance of heavy macromolecules on nuclear volume, dry mass and density, we assembled nuclei in *Xenopus* high-speed (HS) extract, which is obtained by spinning out most organelles, membranes, glycogen, and large protein complexes such as ribosomes. Nuclei grown under these conditions were import- and replication competent (Supplementary Fig. 6m-n, [45,57]). Furthermore, nuclear dry mass was identical to that of control nuclei while nuclear volume was higher (Fig. 5l, m). Interestingly, these nuclei had a higher mass density than the cytoplasmic mass density of HS extract (Fig. 5n, o and Supplementary Notes Fig. 7).

For the simulations, we change the value of the cytoplasmic mass density to $\rho_c = 53.7$ mg mL^{-1} (Fig. 5l). This dramatic change in mass density of proteins compared to control conditions allows us to fine tune the theoretical curve by using different values for the number average molecular weight of protein complexes m_c and m_n. In particular, there is a significant reduction in the value for m_c, which becomes lower than m_n (Supplementary Notes Table 3). This makes sense because the faster spinning applied to obtain HS extract affects the cytoplasmic protein population the most, but has not a big effect in the nucleus because it is able to import the same amount of protein dry mass as control nuclei. As expected from the nuclear dry mass being almost the same as in control experiments, the fitted values for the rates k_{in} and k'_{out} are very similar to the ones obtained from control experiments (Supplementary Notes Table 3). The fitted value for V_{chr} is considerably higher compared to control, but this is most probably caused by the high variability observed in the HS experiments, as evidenced in the large error bars for the experimental data points

(Supplementary Notes Fig. 7). Finally, the fitted κ value is slightly higher compared to control but considering the errors, this difference most likely comes from natural variations.

Taken together, by considering the significant changes in cytoplasmic quantities such as the mass density and number average molecular weight of protein complexes we can match the theoretical nuclear volume and dry mass to the measurements obtained from HS experiments. In particular, the model could reproduce the experimentally observed nuclear mass density, which is *higher* than the cytoplasmic mass density of HS at all time points (Supplementary Notes Fig. 7).

This perturbation shows that spinning out heavy components such as glycogen and ribosomes from the cytoplasm results in a nucleus with a higher mass density than the cytoplasm. These heavy components have a very large molecular weight and greatly contribute to cytoplasmic mass density. However, they are not expected to have significant contributions to colloid osmotic pressure (Figure 5o) because their concentration is not actually high. We then set out to test the effects that osmotic challenges could have in nuclear growth.

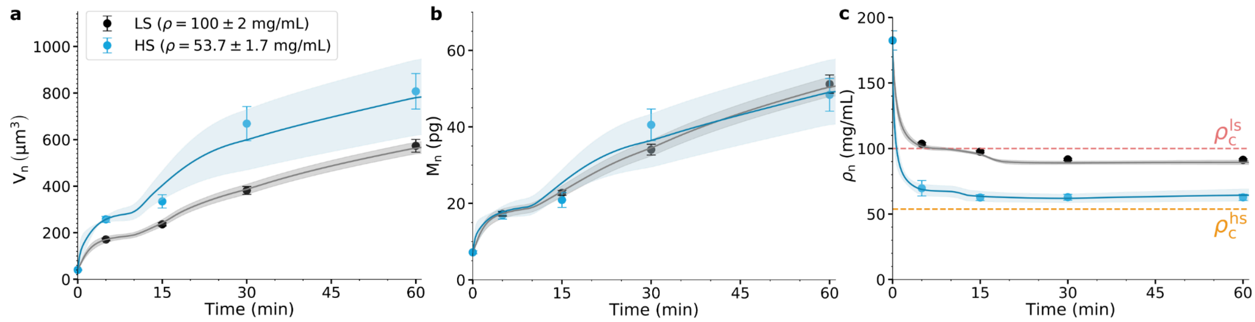

Supplementary Notes Fig. 7 | Simulation of the dynamic model to experiments of nuclei grown in high-speed extract.

(a) Simulation of nuclear volume as a function of time (also depicted in Fig. 5m).

(b) Simulation of nuclear dry mass as a function of time (also depicted in Fig. 5l).

(c) Simulation of nuclear density as a function of time. Dashed lines show the cytoplasmic densities of high-speed extract ($\rho_c^{hs} = 53.7$ mg mL^{-1}) and in control, i.e. low-speed extract ($\rho_c^{ls} = 100$ mg mL^{-1}).

Dots represent experimental data and solid lines are the simulation results. Error bars and shaded areas are

respectively showing the SEM from experiments and simulations. Respective parameters can be found in Supplementary Notes Table 3 (HS condition).

1.4.5 Osmotic shocks

In the following, we investigate nuclear assembly and growth under different types of osmotic shocks and test whether our theory can correctly predict nuclear volume, dry mass and density.

We grew nuclei under various types of osmotic challenges to our *X. laevis* system (Supplementary Notes Fig. 8a). We also plot the nuclear volume and dry mass density measured at t = 60 min along with the respective theoretical predictions against cytoplasmic mass density, which has a value of $\rho_c = 100 \pm 2$ mg mL^{-1} in control (Supplementary Notes Fig. 8b, c). To exert hypo-osmotic shocks, the extract is diluted by addition of CSF-XB buffer ($\rho_c < 100$ mg mL^{-1}) and for hyper-osmotic shocks, a solution of BSA proteins is added to the extract to increase cytoplasmic mass density and protein concentration ($\rho_c > 100$ mg mL^{-1}).

To obtain the respective theoretical predictions we use Supplementary Equation (5) to calculate the steady-state nuclear volumes using the measured nuclear dry mass as input. We assumed the number average mass of cytoplasmic and nuclear protein complexes to remain constant for all osmotic challenges with values of 155 kDa and 131 kDa respectively. To account for hypo-osmotic shocks in our predictions, we use lower values of the cytoplasmic mass density ρ_c (75 and 50 mg mL^{-1}). In the case of hyper-osmotic shocks we assumed a two-component system, where the first component represents cytoplasmic protein complexes with 100 mg mL^{-1} density and the second component represents the BSA solution with a mass density of ρ_{BSA}. Given that BSA proteins are inert and do not form complexes, the average protein mass of this solution is just the respective polypeptide weight of BSA $m_{BSA} = 66$ kDa [58]. Therefore, the total cytoplasmic mass density is $\rho_c + \rho_{BSA}$ and the total cytoplasmic protein concentration is

$\frac{\rho_c}{m_c} + \frac{\rho_{BSA}}{m_{BSA}}$. Chromatin pressure is calculated assuming that we have two copies of the *X. laevis* genome, a persistence length of 150 nm and a chromatin compaction ratio of 40 bp nm^{-1}.

There is an excellent agreement between theory and experiment in the case of control and hypo-osmotic shock data and a reasonably good agreement in the case of hyper-osmotic shock data (Supplementary Notes Fig. 8b, c). The reason for the larger discrepancy in the hyper-osmotic shock data is most likely a consequence of the smaller size and much higher density of nuclei in this case, which makes it a highly non-ideal system. This makes our prediction, which assumes an ideal form for the colloid osmotic pressure, less accurate.

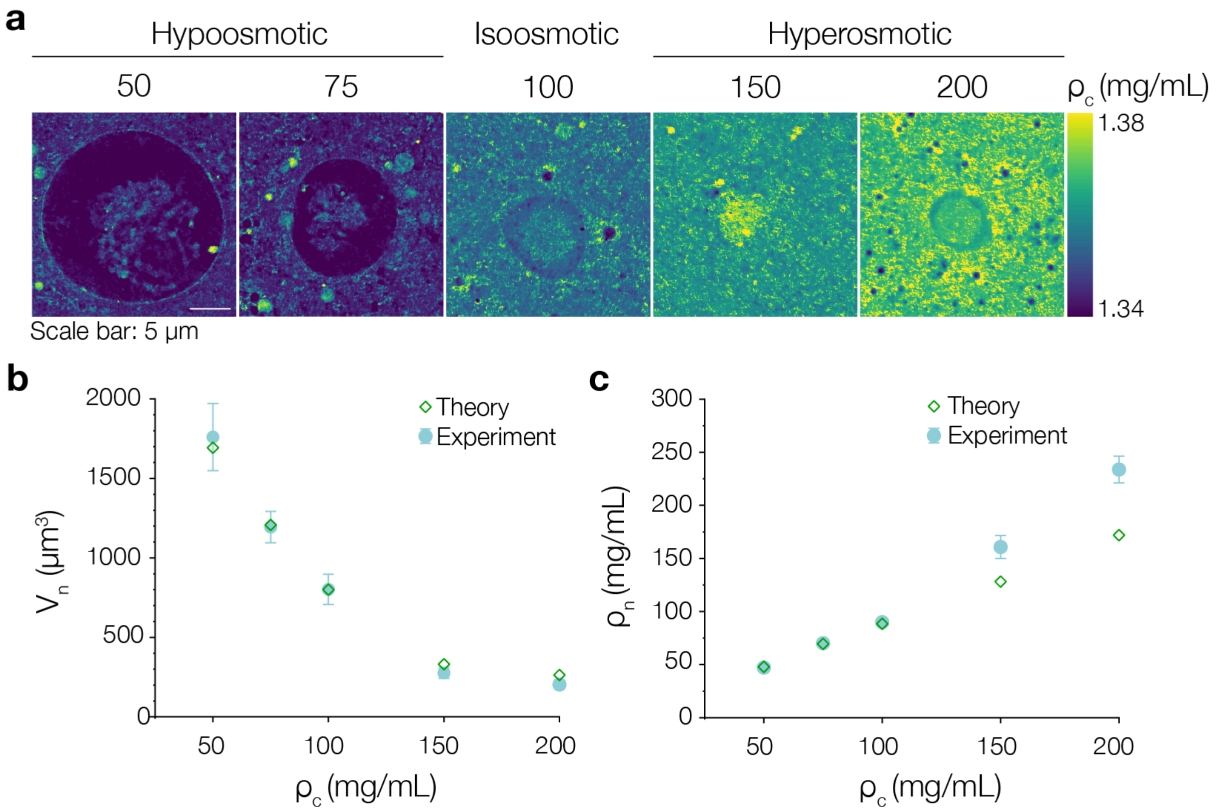

Supplementary Notes Fig. 8 | Nuclei alter their volume in response to changes in total protein density.

(a) Representative RI images of nuclei assembled in *Xenopus* egg extract with reducing protein density by dilution with CSF-XB buffer (Hypotonic) or increasing protein density by adding BSA (Hypertonic). Undiluted extract has a protein concentration of 100 ± 2 mg mL^{-1}. Scale bar: 5 µm. Bar shows RI distribution range.

(b) Quantification of nuclear volumes (V_n) in response to changing protein density. Experimental data shown in blue circles (Mean ± SEM) and theoretical predictions as green diamonds.
(c) Quantification of nuclear density (ρ_n) in response to changing protein density. Experimental data shown in blue circles (Mean ± SEM) and theoretical predictions as green diamonds.

1.5 Technical implementation

In this section, we explain in more detail how the theoretical curves shown in the figures of the main text and here were obtained. We start with the parameter κ that represents chromatin properties. For phase 1, when there is no closed nuclear envelope, we assume the nucleus to be the space occupied by the unfolding chromatin. We describe chromatin as a flexible, self-avoiding polymer. The polymer's radius of gyration depends on κ and number of base pairs via $R_g = \kappa N^{3/5}$ (section 1.1.3). By equating the volume of the sphere with $R = R_g$ to the measured volume $V_n = (4\pi/3)R_g^3$, we thus find the value of $\kappa(t)$ at time t:

$$\kappa(t) = \left(\frac{3\,V_n(t)}{4\pi}\right)^{1/3} N_{bp}^{-3/5} \qquad \text{(Phase 1)}\,, \tag{29}$$

where $V_n(t)$ is the mean nuclear volume measured at time t. To find $\kappa(t)$ in phase 2 of nuclear assembly and growth, we numerically solve the pressure balance equation for $\kappa(t)$:

$$0 = \frac{M_n(t) - M_{chr}(t)}{m_n\left(V_n(t) - V_{chr}\right)} - \frac{\rho_c}{m_c} + \frac{5}{4}\left(\frac{4\pi}{3}\right)^{5/4} \kappa(t)^{15/4} \left(\frac{N_{bp}(t)}{V_n(t)}\right)^{9/4}, \tag{30}$$

where $V_n(t)$ is the mean nuclear volume measured at time t, $M_n(t)$ the mean nuclear dry mass measured at time t, $M_{chr}(t)$ the estimated (calculated) chromatin mass at time t, and $N_{bp}(t)$ the number of base pairs of the genome at time t. The number of proteins in the nucleus was calculated from the experimental data as: $N_n(t) = \left(M_n(t) - M_{chr}(t)\right)/m_n$. Note that $M_{chr}(t)$ is calculated from Supplementary Equation (3) and that both $M_{chr}(t)$ and $N_{bp}(t)$ double their values after replication.

The times at which the experimental measurements were performed are $t = 0,\ 5,\ 15,\ 30$ and 60 min. We define $t^{*} = 10$ min as the time where phase 2 begins and $\kappa^{*} = \kappa(t^{*})$. There are limitations to the values that κ^{*} can take because the corresponding nuclear volume should be smaller than the mean nuclear volume measured at 15 min. The upper bound is then κ_{15}, which is determined by inserting $V_{\mathrm{n}}(t = 15)$ in Supplementary Equation (29). We then choose:

$$\kappa^{*} = f_{\mathrm{k}}\left(\kappa_{15} - \kappa_{5}\right) + \kappa_{5}, \tag{31}$$

where $0 < f_{\mathrm{k}} < 1$ is a simulation parameter that lets us modulate the nuclear volume at the start of phase 2.

We next use κ values at $0,\ 5,\ 15,\ 30,\ 60$ min and at $t^{*} = 10$ min to find a fit function that describes $\kappa(t)$ at any time point:

$$\begin{aligned} \kappa(t) &= \kappa_{1}(t), \quad 0 \le t < t^{*} \\ \kappa(t) &= \kappa_{2}(t), \qquad t > t^{*}, \end{aligned} \tag{32}$$

where $\kappa_{1}(t)$ is a least squares fit function for phase 1 with a Michaelis-Menten saturation curve and $\kappa_{2}(t)$ for phase 2, with a sigmoidal curve.

Once a function for $\kappa(t)$ is obtained, we can use it to solve the system of two ordinary differential equations (ODEs), Supplementary Equations (25) and (26). Given that Supplementary Equation (26) is non-linear, an analytic solution cannot be obtained and we have to solve these equations numerically. For this purpose, we first bring the system of ODEs to a dimensionless form by choosing the following dimensionless units:

$$t \rightarrow \bar{t} = t \cdot k_{\mathrm{in}}, \qquad V_{\mathrm{n}} \rightarrow \overline{V}_{\mathrm{n}} = \frac{V_{\mathrm{n}}}{V_{15}}, \qquad N_{\mathrm{n}} \rightarrow \overline{N}_{\mathrm{n}} = \frac{N_{\mathrm{n}}}{N_{15}},$$

where N_{15}, V_{15} respectively denote the nuclear number of proteins and volume measured at 15 min. With these definitions we can bring other quantities to dimensionless form, for

instance, for the unit of length L, we have $L \to \bar{L} = L/V_{15}^{1/3}$. Therefore, the equations that determine the nuclear protein number and volume during phase 1 become:

$$\bar{V}_{\mathrm{n}}(t) = \frac{4\pi}{3}\,\bar{\kappa}(t)^{3}\,N_{\mathrm{bp}}^{9/5} \tag{33}$$

$$\bar{N}_{\mathrm{n}}(t) = \bar{n}_{\mathrm{c}}\left(\bar{V}_{\mathrm{n}}(t) - \bar{V}_{\mathrm{chr}}\right), \tag{34}$$

where Supplementary Equation (33) follows directly from Supplementary Equation (29) and Supplementary Equation (34) follows from Supplementary Equation (30) without the chromatin pressure term, with $\bar{n}_{\mathrm{c}} = \left(\rho_{\mathrm{c}} V_{15}\right)/\left(m_{\mathrm{c}} N_{15}\right)$. For phase 2, the ODEs have the following dimensionless form:

$$\frac{d\bar{N}_{\mathrm{n}}(\bar{t})}{d\bar{t}} = \bar{V}_{\mathrm{n}}(\bar{t})\,\bar{n}_{\mathrm{c}} - \bar{k}\,\bar{V}_{\mathrm{n}}(\bar{t})\frac{\bar{N}_{\mathrm{n}}(\bar{t})}{\nu(\bar{t})} \tag{35}$$

$$\frac{\bar{N}_{\mathrm{n}}(\bar{t})}{\bar{V}_{\mathrm{n}}(\bar{t}) - \bar{V}_{\mathrm{chr}}} - \bar{n}_{\mathrm{c}} + \frac{5}{4N_{15}}\left(\frac{4\pi}{3}\right)^{5/4}\bar{\kappa}(\bar{t})^{15/4}\left(\frac{N_{\mathrm{bp}}(\bar{t})}{\bar{V}_{\mathrm{n}}(\bar{t})}\right)^{9/4} = 0\,, \tag{36}$$

where $\bar{k} = k'_{\mathrm{out}} V_{15}/k_{\mathrm{in}}$.

1.5.1 Stochasticity of phase 2 starting time point

Our model includes phases 1 and 2 of nuclear growth, where nuclear volume and protein number are determined by a different set of equations, see Supplementary Equations (33) and (34) for phase 1 and (35) and (36) for phase 2. We defined a time t^{*} where the model switches from the phase 1 equations to the phase 2 equations and assumed a value of $t^{*} = 10$ min based on experimental observations. In reality, the start of phase 2 is somewhat different for each nucleus grown in our system and we included this effect in the following way: for each fitted set of parameters, we performed multiple simulations, where we shifted the phase 2 starting point of $t^{*} = 10$ min by a random Gaussian number with mean 0 and a variance of 1 minute. The same shift is applied to the starting and end times of replication. This will of course change the fitted function $\kappa(t)$, but the rest of the

parameter values are left unchanged. We perform $N_{\mathrm{stoch}} = 50$ such stochastic trials and the resulting average curve is shown in the figures for the respective data sets.

1.5.2 Choice of the excluded volume of chromatin

In section 1.1.5, we introduced the parameter V_{chr}, which represents the chromatin excluded volume in the nucleus. This quantity is in principle time-dependent because the parameter $\kappa(t)$, which is a measure of chromatin condensation, is also time dependent. This would make V_{chr} another system variable like nuclear volume $V_{\mathrm{n}}(t)$ and protein number $N_{\mathrm{n}}(t)$. This poses a problem because we only have two equations describing our system, see Supplementary Equations (35) and (36).

For phase 1, we proceed as follows: at $t = 0$, we choose the value of V_{chr}, for which we obtain the experimentally measured volume and dry mass under the assumption of an osmotic balance. The equation determining V_{chr} is then:

$$\frac{M_{\mathrm{n}}(0)}{m_{\mathrm{n}}\left(V_{\mathrm{n}}(0) - V_{\mathrm{chr},0}\right)} - \frac{\rho_{\mathrm{c}}}{m_{\mathrm{c}}} = 0, \tag{37}$$

from which it follows:

$$V_{\mathrm{chr},0} = V_{\mathrm{n}}(0) - \frac{M_{\mathrm{n}}(0)\, m_{\mathrm{c}}}{m_{\mathrm{n}}\, \rho_{\mathrm{c}}}, \tag{38}$$

where $V_{\mathrm{n}}(0)$, $M_{\mathrm{n}}(0)$ respectively denote the mean nuclear volume and dry mass measured at $t = 0$. We then change the value of V_{chr} from $V_{\mathrm{chr},0}$ to a different value, which we define as $V_{\mathrm{chr},5}$, at $t = 5$ min in a linear fashion. For the rest of the simulation, we will assume $V_{\mathrm{chr}}(t) = V_{\mathrm{chr},5}$. Therefore, $V_{\mathrm{chr}}(t)$ has the following form:

$$\begin{aligned} V_{\mathrm{chr}}(t) &= V_{\mathrm{chr},0} + \frac{V_{\mathrm{chr},5} - V_{\mathrm{chr},0}}{5\ \mathrm{min}}\, t, \quad 0 \le t < 5\ \mathrm{min} \\ V_{\mathrm{chr}}(t) &= V_{\mathrm{chr},5}, \qquad t \ge 5\ \mathrm{min}. \end{aligned} \tag{39}$$

To choose the value of $V_{chr,5}$, we first calculate V_{chr} using Supplementary Equation (38) and the experimental data for V_n and M_n for all time points. This gives us the upper bound estimate for the V_{chr} under the assumption of zero chromatin pressure. We then set $V_{chr,5}$ equal to the smallest calculated V_{chr}. The reason for this is to ensure that nuclear colloid osmotic pressure is less or equal to cytoplasmic colloid osmotic pressure at all times in the simulations, so that the pressure balance including the contribution of chromatin could be fulfilled. In the majority of cases, the smallest value is indeed just the value calculated for $t = 5$ min, but in some rare cases we have to use the value provided by the formula that corresponds to later times.

1.5.3 Validity of the fitted chromatin parameter values

The chromatin parameter is defined as $\kappa = l_p^{2/5}/\rho_{chr}^{3/5}$ and combines the chromatin persistence length and compaction ratio. To check whether our fitted κ values correspond to the persistence length and compaction ratio values reported in the literature (Supplementary Notes Table 2), we plot in Supplementary Notes Fig. 9 a curve on the plane spanned by the chromatin compaction ratio and persistence length, which corresponds to the average value of fitted κ at t = 60 min in control experiments, with the corresponding error range (defined by the standard error of mean). The axis limits correspond to the ranges reported in the literature for the chromatin compaction ratio and persistence length (Supplementary Notes Table 2), which shows that our fitted κ lies well within the expected range reported in the literature.

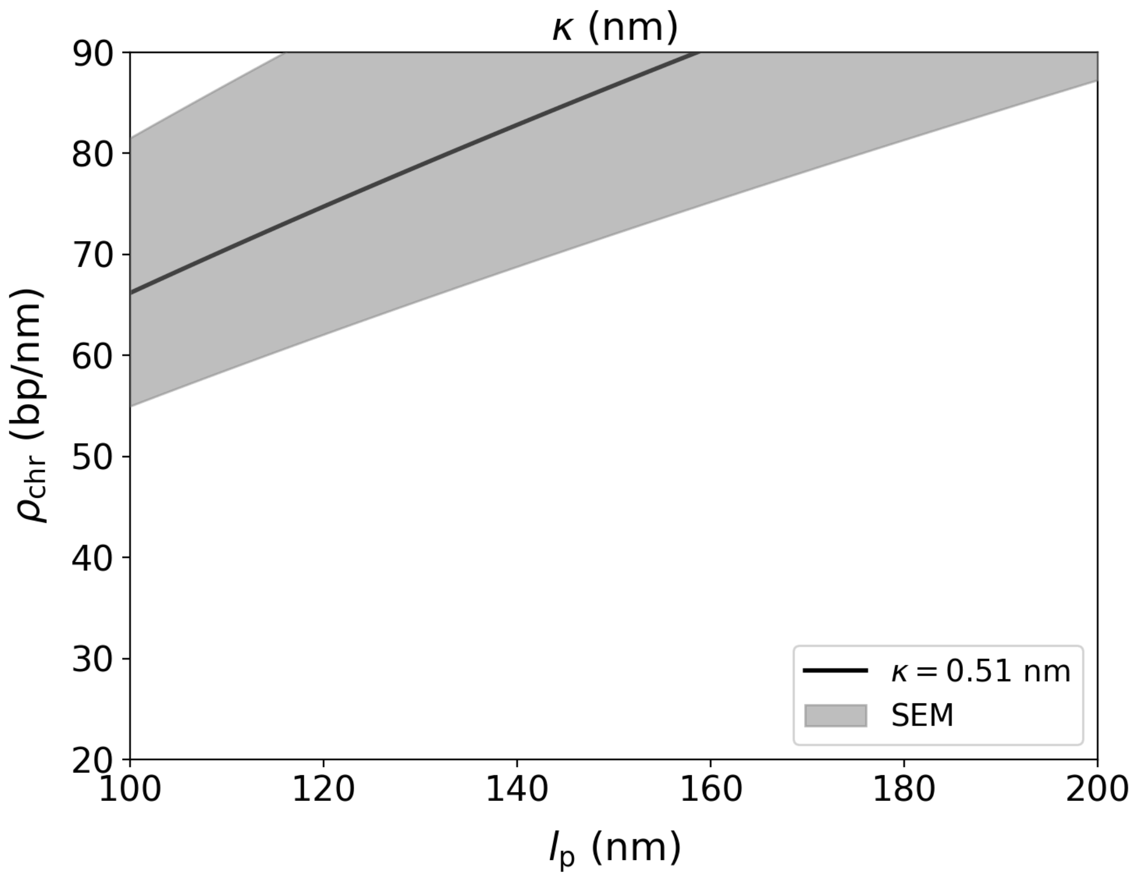

Supplementary Notes Fig. 9 | The fitted value of the chromatin parameter is in the expected physiological range.

The curve represents a specific value of the chromatin parameter $\kappa = l_p^{2/5}/\rho_{chr}^{3/5}$ on the plane spanned by the chromatin persistence length l_p and the chromatin compaction ratio ρ_{chr}. The axis ranges reflect the values reported in the literature (Supplementary Notes Table 2). The specific value $\kappa = 0.51\ \mathrm{nm}$ is the average of the experimentally expected values of κ at 60 min, obtained from control experiments (Supplementary Notes Table 3). The shaded area denotes the respective standard error of mean (SEM).

1.5.4 Variability in experiments and of simulation parameters

We remarked in section 1.5.1 that the start of phase 2 may vary for nuclei assembled in our experiments. This variability is also present in other important quantities such as the nuclear volume and dry mass. To reproduce this variability also in simulations (and thus gain information on the variability of model parameters), we fitted the theory to each

individual experimental data set (for the example shown below, an individual data set is a set of control measurements performed for every perturbation experiment) and then take the average of the obtained theoretical curves, which will also provide us with an error for the theoretical predictions. As we can see in Supplementary Notes Figs. 10, 11 (left panels), the different (control) data sets show significant variability in volume and dry mass, which is reflected in the gray area representing the standard error of mean (right panels) in the theory plot.

To produce theoretical curves with error bars for the perturbation conditions, we use individual biological replicates as individual data sets. This is how we generated all the plots here and in the main text. For such theoretical fits we can now provide the parameter values with the respective error (SEM) which would indicate variability necessary to capture the natural variability present in the experimental system (Supplementary Notes Table 3).

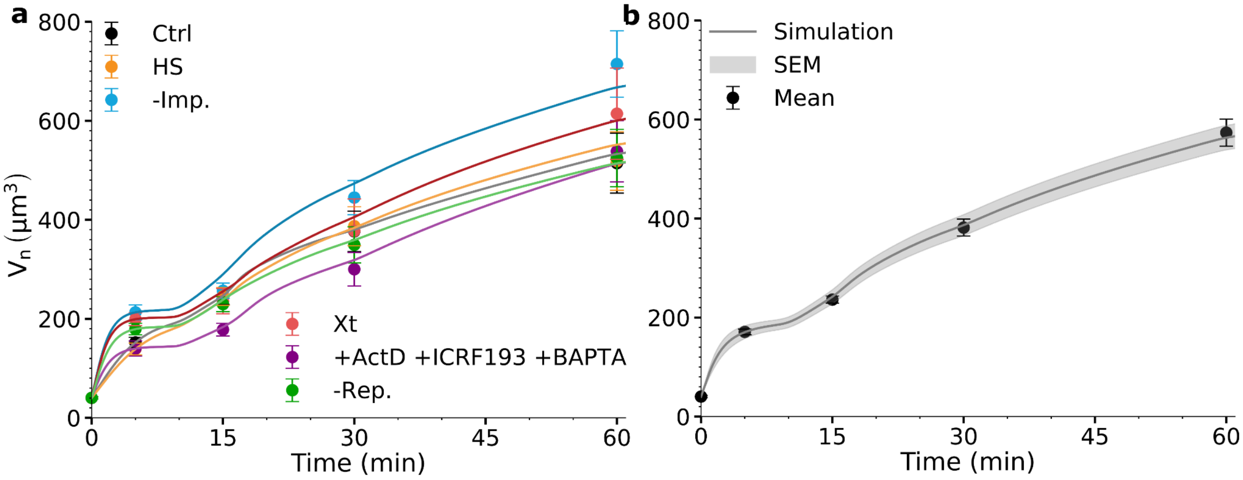

Supplementary Notes Fig. 10 | Variability of nuclear volume measurements in control conditions and respective theoretical fits.

(a) Nuclear volume measured from the control measurements of the following experiments: no perturbation (Ctrl), nuclei grown in high-speed extract (HS), nuclear import inhibition (-Imp.), nuclei grown from X. tropicalis sperm chromatin (Xt), inhibition of chromatin decondensation and nuclear import (+ActD +ICRF193 +BAPTA) and inhibition of DNA replication (-Rep.). Experimental data is shown as dots with error bars (SEM). Individual theory fits for each experiment are shown as solid lines.

(b) Nuclear volume obtained from the average of all control experiments. The black dots are the average of all the experimental data from a. The black line is obtained by averaging the theory fits from a and the

shaded region depicts the respective SEM.

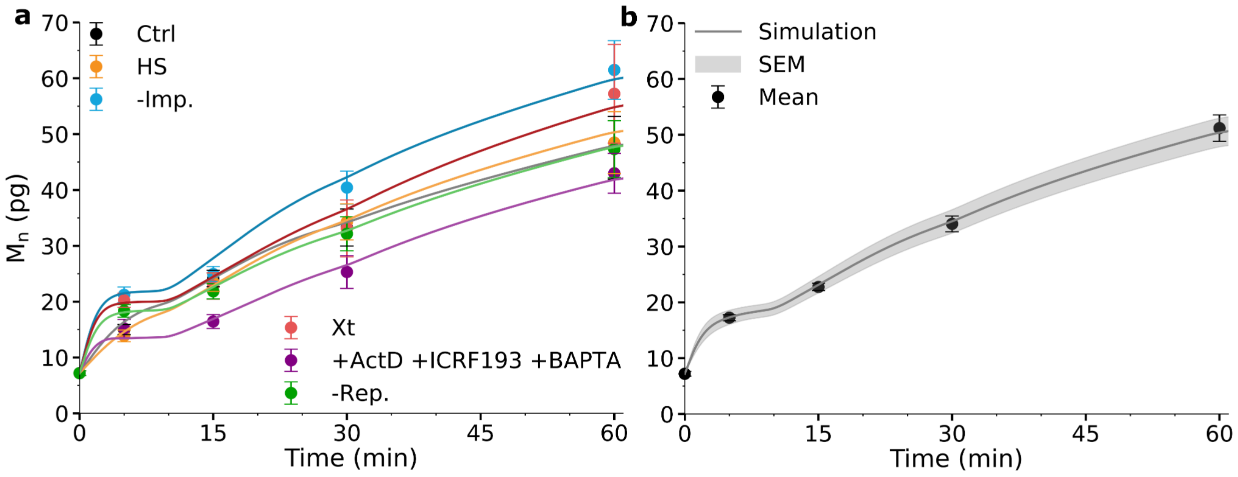

Supplementary Notes Fig. 11 | Variability of nuclear dry mass measurements in control conditions and respective theoretical fits.

(a) Nuclear dry mass measured from the control measurements of the following experiments: no perturbation (Ctrl), nuclei grown in high-speed extract (HS), nuclear import inhibition (-Imp.), nuclei grown from X. tropicalis sperm chromatin (Xt), inhibition of chromatin decondensation and nuclear import (+ActD +ICRF193 +BAPTA) and inhibition of DNA replication (-Rep.). Experimental data is shown as dots with error bars (SEM). Individual theory fits for each experiment are shown as solid lines.

(b) Nuclear dry mass obtained from the average of all control experiments. The black dots are the average of all the experimental data from a. The black line is obtained by averaging the theory fits from a and the shaded region depicts the respective SEM.

References

1. Deviri, D. & Safran, S. A. Balance of osmotic pressures determines the nuclear-to-cytoplasmic volume ratio of the cell. *Proc. Natl. Acad. Sci. U. S. A.* 119, e2118301119 (2022).
2. Lemière, J., Real-Calderon, P., Holt, L. J., Fai, T. G. & Chang, F. Control of nuclear size by osmotic forces in Schizosaccharomyces pombe. *Elife* 11, (2022).
3. Rollin, R., Joanny, J.-F. & Sens, P. Physical basis of the cell size scaling laws. *Elife* 12, (2023).
4. Cadart, C., Venkova, L., Piel, M. & Cosentino Lagomarsino, M. Volume growth in animal cells is cell cycle dependent and shows additive fluctuations. *Elife* 11, e70816 (2022).
5. Milo, R. & Phillips, R. *Cell Biology by the Numbers*. (CRC Press, Boca Raton, FL, 2015).
6. Kim, D.-H. *et al.* Volume regulation and shape bifurcation in the cell nucleus. *J. Cell Sci.* 128, 3375–3385 (2015).
7. Bohnsack, M. T., Stüven, T., Kuhn, C., Cordes, V. C. & Görlich, D. A selective block of nuclear actin export stabilizes the giant nuclei of Xenopus oocytes. *Nat. Cell Biol.* 8, 257–263 (2006).
8. Mitchison, T. J. Colloid osmotic parameterization and measurement of subcellular crowding. *Mol. Biol. Cell* 30, 173–180 (2019).
9. Pennacchio, F. A. *et al.* Force-biased nuclear import sets nuclear-cytoplasmic volumetric coupling by osmosis. *bioRxiv* (2022) doi:10.1101/2022.06.07.494975.
10. Frey, S. *et al.* Surface properties determining passage rates of proteins through nuclear pores. *Cell* 174, 202–217 (2018).
11. Wühr, M. *et al.* The nuclear proteome of a vertebrate. *Curr. Biol.* 25, 2663–2671 (2015).
12. Wang, C.-H., Mehta, P. & Elbaum, M. Thermodynamic paradigm for solution demixing inspired by nuclear transport in living cells. *Phys. Rev. Lett.* 118, 158101 (2017).
13. Cautain, B., Hill, R., de Pedro, N. & Link, W. Components and regulation of nuclear transport

processes. *FEBS J.* 282, 445–462 (2015).

14. Fried, H. & Kutay, U. Nucleocytoplasmic transport: taking an inventory. *Cell. Mol. Life Sci.* 60, 1659–1688 (2003).
15. Nobel, P. The Boyle-van't Hoff relation. *J. Theor. Biol.* 23, 375–379 (1969).
16. Milo, R. What is the total number of protein molecules per cell volume? A call to rethink some published values: Insights & Perspectives. *Bioessays* 35, 1050–1055 (2013).
17. Biswas, A., Kim, K., Cojoc, G., Guck, J. & Reber, S. The Xenopus spindle is as dense as the surrounding cytoplasm. *Dev. Cell* 56, 967–975.e5 (2021).
18. Nucleic acids sizes and molecular weights & s - Various - BNID 110968. https://bionumbers.hms.harvard.edu/bionumber.aspx?id=110968&ver=2&trm=110968&org=.
19. Halverson, J. D., Smrek, J., Kremer, K. & Grosberg, A. Y. From a melt of rings to chromosome territories: the role of topological constraints in genome folding. *Rep. Prog. Phys.* 77, 022601 (2014).
20. Rubinstein, M. & Colby, R. H. *Polymer Physics*. (Oxford University Press, London, England, 2003).
21. Fudenberg, G. *et al.* Formation of chromosomal domains by loop extrusion. *Cell Rep.* 15, 2038–2049 (2016).
22. Cook, P. R. & Marenduzzo, D. Entropic organization of interphase chromosomes. *J. Cell Biol.* 186, 825–834 (2009).
23. Boettiger, A. N. *et al.* Super-resolution imaging reveals distinct chromatin folding for different epigenetic states. *Nature* 529, 418–422 (2016).
24. Cacciuto, A. & Luijten, E. Self-avoiding flexible polymers under spherical confinement. *Nano Lett.* 6, 901–905 (2006).

25. G., D. G. P. Scaling Concepts in Polymer Physics. (1979).

26. Sugawara, T. & Kimura, A. Physical properties of the chromosomes and implications for development. *Dev. Growth Differ.* 59, 405–414 (2017).

27. Dekker, J. & Steensel, B. The Spatial Architecture of Chromosomes. *Handbook of Systems Biology* 137–151 (2013).

28. Bystricky, K., Heun, P., Gehlen, L., Langowski, J. & Gasser, S. M. Long-range compaction and flexibility of interphase chromatin in budding yeast analyzed by high-resolution imaging techniques. *Proc. Natl. Acad. Sci. U. S. A.* 101, 16495–16500 (2004).

29. Efremov, A. K., Hovan, L. & Yan, J. Nucleus size and its effect on nucleosome stability in living cells. *Biophys. J.* 121, 4189–4204 (2022).

30. Tripathi, K. & Menon, G. I. Chromatin compaction, auxeticity, and the epigenetic landscape of stem cells. *Phys. Rev. X.* 9, (2019).

31. Kopito, R. B. & Elbaum, M. Reversibility in nucleocytoplasmic transport. *Proc. Natl. Acad. Sci. U. S. A.* 104, 12743–12748 (2007).

32. Kopito, R. B. & Elbaum, M. Nucleocytoplasmic transport: a thermodynamic mechanism. *HFSP J.* 3, 130–141 (2009).

33. Görlich, D., Seewald, M. J. & Ribbeck, K. Characterization of Ran-driven cargo transport and the RanGTPase system by kinetic measurements and computer simulation. *EMBO J.* 22, 1088–1100 (2003).

34. Andreu, I. *et al.* Mechanical force application to the nucleus regulates nucleocytoplasmic transport. *Nat. Cell Biol.* 24, 896–905 (2022).

35. Lee, S. J., Matsuura, Y., Liu, S. M. & Stewart, M. Structural basis for nuclear import complex dissociation by RanGTP. *Nature* 435, 693–696 (2005).

36. Bierbaum, M. & Bastiaens, P. I. H. Cell cycle-dependent binding modes of the ran exchange factor RCC1 to chromatin. *Biophys. J.* 104, 1642–1651 (2013).
37. Nemergut, M. E. & Macara, I. G. Nuclear import of the ran exchange factor, RCC1, is mediated by at least two distinct mechanisms. *J. Cell Biol.* 149, 835–850 (2000).
38. Görlich, D. *et al.* Importin provides a link between nuclear protein import and U snRNA export. *Cell* 87, 21–32 (1996).
39. Görlich, D., Panté, N., Kutay, U., Aebi, U. & Bischoff, F. R. Identification of different roles for RanGDP and RanGTP in nuclear protein import. *EMBO J.* 15, 5584–5594 (1996).
40. Kim, S. & Elbaum, M. A simple kinetic model with explicit predictions for nuclear transport. *Biophys. J.* 105, 565–569 (2013).
41. Riddick, G. & Macara, I. G. A systems analysis of importin-{alpha}-{beta} mediated nuclear protein import. *J. Cell Biol.* 168, 1027–1038 (2005).
42. Smith, A. E., Slepchenko, B. M., Schaff, J. C., Loew, L. M. & Macara, I. G. Systems analysis of Ran transport. *Science* 295, 488–491 (2002).
43. Furuta, M., Hori, T. & Fukagawa, T. Chromatin binding of RCC1 during mitosis is important for its nuclear localization in interphase. *Mol. Biol. Cell* 27, 371–381 (2016).
44. Chen, P., Mishra, S., Prabha, H., Sengupta, S. & Levy, D. L. Nuclear growth and import can be uncoupled. *Mol. Biol. Cell* 35, ar1 (2024).
45. Bernis, C. & Forbes, D. Analysis of nuclear reconstitution, nuclear envelope assembly, and nuclear pore assembly using Xenopus in vitro assays. *Methods Cell Biol.* 122, 165–191 (2014).
46. Levy, D. L. & Heald, R. Nuclear size is regulated by importin α and Ntf2 in Xenopus. *Cell* 143, 288–298 (2010).

47. Hellsten, U. *et al.* The genome of the Western clawed frog Xenopus tropicalis. *Science* 328, 633–636 (2010).

48. UniProt. https://www.uniprot.org/uniprotkb/P06898/entry.

49. UniProt. https://www.uniprot.org/uniprotkb/P02281/entry.

50. UniProt. https://www.uniprot.org/uniprotkb/P84233/entry.

51. UniProt. https://www.uniprot.org/uniprotkb/P62799/entry.

52. UniProt. https://www.uniprot.org/uniprotkb/P15308/entry.

53. Zhou, Z., Yan, R., Jiang, W. & Irudayaraj, J. M. K. Chromatin hierarchical branching visualized at the nanoscale by electron microscopy. *Nanoscale Adv.* 3, 1019–1028 (2021).

54. Wagstaff, K. M., Sivakumaran, H., Heaton, S. M., Harrich, D. & Jans, D. A. Ivermectin is a specific inhibitor of importin α/β-mediated nuclear import able to inhibit replication of HIV-1 and dengue virus. *Biochem. J.* 443, 851–856 (2012).

55. Liashkovich, I. *et al.* Clathrin inhibitor Pitstop-2 disrupts the nuclear pore complex permeability barrier. *Sci. Rep.* 5, (2015).

56. Glover, T. W., Berger, C., Coyle, J. & Echo, B. DNA polymerase alpha inhibition by aphidicolin induces gaps and breaks at common fragile sites in human chromosomes. *Hum. Genet.* 67, 136–142 (1984).

57. Galy, V. *et al.* A role for gp210 in mitotic nuclear-envelope breakdown. *J. Cell Sci.* 121, 317–328 (2008).

58. Albumin aus Rinderserum. https://www.sigmaaldrich.com/DE/de/product/sigma/a2153.
