# Supplementary material for: Conserved nucleocytoplasmic density homeostasis drives cellular organization across eukaryotes
Source: Nat Commun. 2025 Aug 15;16:7597. doi: 10.1038/s41467-025-62605-0 (PMC12356907; doi:10.1038/s41467-025-62605-0)
Supplement: Supplementary file 2 — Description of Additional Supplementary Information [file 41467_2025_62605_MOESM2_ESM.pdf]

Description of Additional Supplementary Files

File Name: Supplementary Movie 1
Description: A dynamic model for nuclear assembly and growth in Xenopus egg extracts captures the evolution of nuclear volume (Vn) over time in both control and perturbed nuclei (-Import, - Replication, X. tropicalis). Video: 10.6084/m9.figshare.23668305

File Name: Supplementary Movie 2
Description: First embryonic divisions in a wildtype (left) and tetraploid (right) C. elegans embryo imaged by ODT. Scale bar = 20 µm. The RI range in the movie is 1.34-1.41. Images were acquired at 1-minute intervals. Video: 10.6084/m9.figshare.28045058
