# Supplementary material for: Conserved nucleocytoplasmic density homeostasis drives cellular organization across eukaryotes
Source: Nat Commun. 2025 Aug 15;16:7597. doi: 10.1038/s41467-025-62605-0 (PMC12356907; doi:10.1038/s41467-025-62605-0)
Supplement: Supplementary file 5 — Reporting Summary [file 41467_2025_62605_MOESM5_ESM.pdf]

nature portfolio

Corresponding author(s): Simone Reber
Last updated by author(s): Jun 23, 2025

Reporting Summary

Nature Portfolio wishes to improve the reproducibility of the work that we publish. This form provides structure for consistency and transparency in reporting. For further information on Nature Portfolio policies, see our Editorial Policies and the Editorial Policy Checklist.

Statistics

For all statistical analyses, confirm that the following items are present in the figure legend, table legend, main text, or Methods section.

n/a	Confirmed	
☐	☒	The exact sample size (*n*) for each experimental group/condition, given as a discrete number and unit of measurement
☐	☒	A statement on whether measurements were taken from distinct samples or whether the same sample was measured repeatedly
☐	☒	The statistical test(s) used AND whether they are one- or two-sided<br>*Only common tests should be described solely by name; describe more complex techniques in the Methods section.*
☒	☐	A description of all covariates tested
☐	☒	A description of any assumptions or corrections, such as tests of normality and adjustment for multiple comparisons
☐	☒	A full description of the statistical parameters including central tendency (e.g. means) or other basic estimates (e.g. regression coefficient) AND variation (e.g. standard deviation) or associated estimates of uncertainty (e.g. confidence intervals)
☐	☒	For null hypothesis testing, the test statistic (e.g. *F*, *t*, *r*) with confidence intervals, effect sizes, degrees of freedom and *P* value noted<br>*Give P values as exact values whenever suitable.*
☒	☐	For Bayesian analysis, information on the choice of priors and Markov chain Monte Carlo settings
☒	☐	For hierarchical and complex designs, identification of the appropriate level for tests and full reporting of outcomes
☐	☒	Estimates of effect sizes (e.g. Cohen's *d*, Pearson's *r*), indicating how they were calculated

Our web collection on statistics for biologists contains articles on many of the points above.

Software and code

Policy information about availability of computer code

Data collection	Tomogram field retrieval and reconstruction: https://github.com/OpticalDiffractionTomography/ODT_FieldTomogramGUI<br>Tomogram acquisition: https://github.com/OpticalDiffractionTomography/ODT_Image_Acquisition<br>Fluorescence images were acquired using MicroManager or Nikon Elements AR
Data analysis	GraphPad Prism version 9.0 for statistical analysis, FIJI version 2.14.0/1.54f and Matlab R2019b for image analysis,

For manuscripts utilizing custom algorithms or software that are central to the research but not yet described in published literature, software must be made available to editors and reviewers. We strongly encourage code deposition in a community repository (e.g. GitHub). See the Nature Portfolio guidelines for submitting code & software for further information.

Data

Policy information about availability of data

All manuscripts must include a data availability statement. This statement should provide the following information, where applicable:

- Accession codes, unique identifiers, or web links for publicly available datasets
- A description of any restrictions on data availability
- For clinical datasets or third party data, please ensure that the statement adheres to our policy

Manuscript has a data availability statement.

Research involving human participants, their data, or biological material

Policy information about studies with human participants or human data. See also policy information about sex, gender (identity/presentation), and sexual orientation and race, ethnicity and racism.

Reporting on sex and gender	n.a.
Reporting on race, ethnicity, or other socially relevant groupings	n.a.
Population characteristics	n.a.
Recruitment	n.a.
Ethics oversight	n.a.

Note that full information on the approval of the study protocol must also be provided in the manuscript.

Field-specific reporting

Please select the one below that is the best fit for your research. If you are not sure, read the appropriate sections before making your selection.

☒ Life sciences ☐ Behavioural & social sciences ☐ Ecological, evolutionary & environmental sciences

For a reference copy of the document with all sections, see nature.com/documents/nr-reporting-summary-flat.pdf

Life sciences study design

All studies must disclose on these points even when the disclosure is negative.

Sample size	Sample size was not pre determined by statistical methods before the execution of the experiments.
Data exclusions	No data was excluded.
Replication	All experiments were replicated independently, the number of replicates has been indicated in each figure legend.
Randomization	Yes, cells and nuclei were measured randomly.
Blinding	No blinding was carried out as all measurements were quantitative and were not biased by subjective perception.

Reporting for specific materials, systems and methods

We require information from authors about some types of materials, experimental systems and methods used in many studies. Here, indicate whether each material, system or method listed is relevant to your study. If you are not sure if a list item applies to your research, read the appropriate section before selecting a response.

Materials & experimental systems

n/a	Involved in the study	
☐	☒	Antibodies
☐	☒	Eukaryotic cell lines
☒	☐	Palaeontology and archaeology
☐	☒	Animals and other organisms
☒	☐	Clinical data
☒	☐	Dual use research of concern
☒	☐	Plants

Methods

n/a	Involved in the study	
☒	☐	ChIP-seq
☒	☐	Flow cytometry
☒	☐	MRI-based neuroimaging

Antibodies

Antibodies used	Npm2 antibody was purified as described in reference 38, RPS17 and RPL18 antibodies (HPA055060-100UL and HPA046572-100UL) respectively were commercially obtained.
Validation	No new antibodies were generated. Npm2 was validated as described in reference 38 and by blotting against purified egg and recombinant protein, Ribosomal subunit antibodies were validated by the Human protein Atlas Project.

Eukaryotic cell lines

Policy information about cell lines and Sex and Gender in Research

Cell line source(s)	C. reinhardtii (CC125 Chlamydomonas Resource Center), S cerevisiae (ATCC 204508), C. elegans (N2 and SP346 CGC), D. melanogaster (RRID:CVCL_Z831 DGRC Stock 150), D. rerio (AB strain EZRC), X. laevis (XL-177 kerafast), M. musculus (R1/E ATCC SCRC-1036), H. sapiens (HEK293 ATCC CRL-1573 and hTERT RPE-1 ATCC CRL-4000), All other cell lines used in this study were gifts from colleagues as described in the methods and acknowledgment sections.
Authentication	Cell lines were not authenticated.
Mycoplasma contamination	Cell lines were tested routinely for mycoplasma contaimination.
Commonly misidentified lines (See ICLAC register)	No commonly misidentified cell lines were used in this study.

Animals and other research organisms

Policy information about studies involving animals; ARRIVE guidelines recommended for reporting animal research, and Sex and Gender in Research

Laboratory animals	Adult Xenopus frogs (laevis and tropicalis) were used
Wild animals	n.a.
Reporting on sex	Only adult egg laying females were used for extract preparation. Males were used for sperm isolation.
Field-collected samples	n.a.
Ethics oversight	All experimental protocols involving frogs were performed in accordance with national regulatory standards and ethical rules and reviewed and approved by the LaGeSo under Reg.-Nr. Reg 0113/20.

Note that full information on the approval of the study protocol must also be provided in the manuscript.

Plants

Seed stocks	n.a.
Novel plant genotypes	n.a.
Authentication	n.a.
