# Supplementary figures and images for: Conserved nucleocytoplasmic density homeostasis drives cellular organization across eukaryotes
Source: Nat Commun. 2025 Aug 15;16:7597. doi: 10.1038/s41467-025-62605-0 (PMC12356907; doi:10.1038/s41467-025-62605-0)

Figure 2d

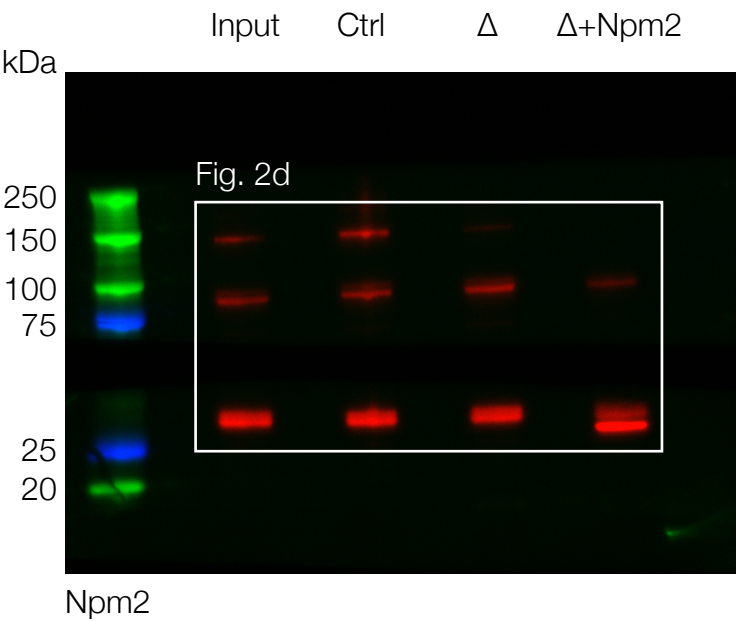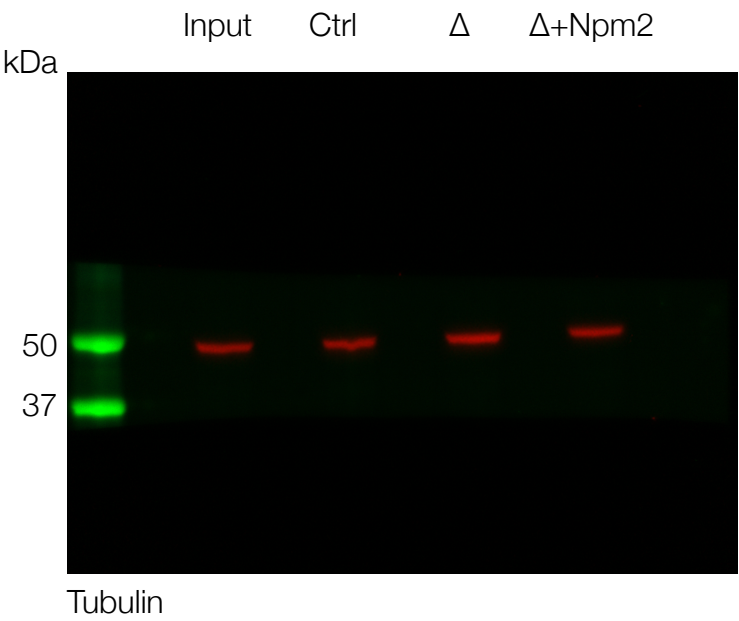

Supplementary Figure 2a

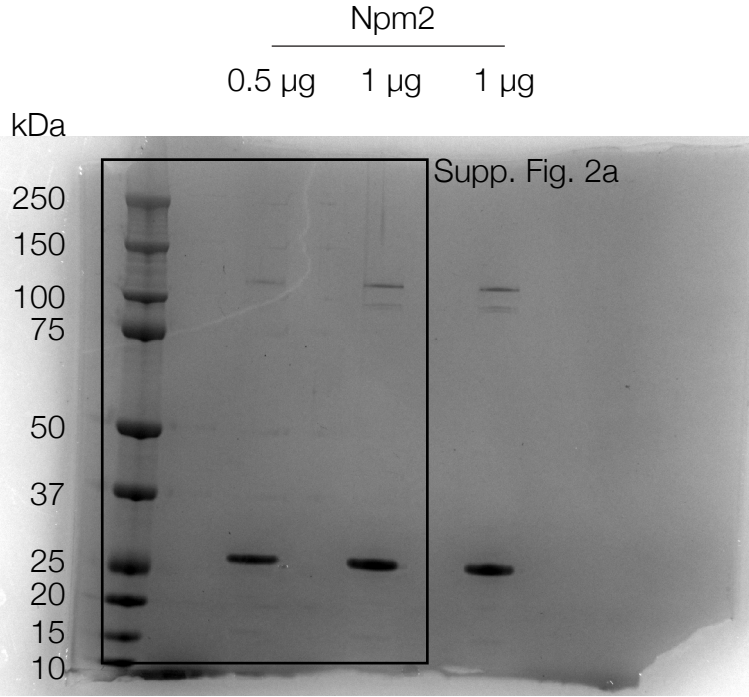

Supplement: Supplementary file 7 — Source Data [file 41467_2025_62605_MOESM7_ESM.zip › blots.pdf]
